# Submolecular modulation of PIEZO1 mechanotransduction with wireless tailor-made nanoswitches

**DOI:** 10.1016/j.bioactmat.2026.03.051

**Published:** 2026-04-27

**Authors:** Simão P.B. Teixeira, Alberto Pardo, Pablo Taboada, Brent M. Bijonowski, Mariana T. Carvalho, Jana B. Nieder, Maja Wolleb, Jess G. Snedeker, Manuela E. Gomes, Rui M.A. Domingues

**Affiliations:** a International Iberian Nanotechnology Laboratory (INL), Avenida Mestre José Veiga s/n, 4715-330, Braga, Portugal; b 3B’s Research Group, Research Institute on Biomaterials, Biodegradables and Biomimetics (I3Bs) of the University of Minho, Headquarters of the European Institute of Excellence on Tissue Engineering and Regenerative Medicine, AvePark – Parque de Ciência e Tecnologia, Rua Ave 1, Edifício 1 (Sede), 4805-694, Barco GMR, Portugal; c ICVS/3B’s–PT Government Associate Laboratory, Braga/Guimarães, Portugal; d Colloids and Polymers Physics Group, Particle Physics Department, Materials Institute (iMATUS), Health Research Institute (IDIS), University of Santiago de Compostela, 15782, Santiago de Compostela, Spain; e Department of Orthopaedics, University Hospital Balgrist, University of Zurich, Lengghalde 5, CH-8008, Zurich, Switzerland; f Institute for Biomechanics, ETH Zurich, CH-8093, Zurich, Switzerland; g Unit for Multidisciplinary Research in Biomedicine (UMIB), School of Medicine and Biomedical Sciences (ICBAS) of the University of Porto, Rua de Jorge de Viterbo Ferreira, 228, 4050-313, Porto, Portugal

**Keywords:** Magnetic therapies, Mechanobiology, Molecular imprinting, PIEZO channels, Stem cells

## Abstract

PIEZO mechanoreceptors play critical roles in fundamental physiological processes such as proprioception or musculoskeletal biomechanics. However, their complex gating mechanisms and downstream signaling are still not completely understood, mainly due to the lack of effective probing tools. Here, we combine molecular imprinting and magnetic concepts to develop tailor-made nanoswitches enabling wireless targeted actuation of PIEZO1. Two epitopes selected *in silico* from distinct domains of PIEZO1 were used as templates for synthesizing magnetically responsive molecularly imprinted nanoparticles. These nanoswitches showed sub-nanomolar affinity for their respective epitope and recognized PIEZO1 in endothelial cells, similarly to antibodies. Applying magnetic fields to actuate PIEZO1 through nanoswitches led to increased calcium signaling, Yes-associated protein (YAP) nuclear translocation, and significant changes in the expression of genes related to PIEZO1 activity, implying that they can transduce the stimulus into intracellular signaling effects. Finally, this wireless actuation system proved to be effective in differentially modulating the behavior of mesenchymal stem cells. Remarkably, the selective targeting of each epitope led to contrasting downstream signaling cascades, implying distinct roles for each superstructure domain in the sophisticated function of these channels. Overall, this technology constitutes a promising tool for studying PIEZO-mediated mechanobiology, opening perspectives for harnessing its potential toward therapeutic approaches.

## 1 Introduction

Mechanotransduction is the centrally important ability of cells to sense physical cues, such as extracellular matrix (ECM) stiffness, microarchitecture, shear or compressive forces, and transduce them into intracellular biochemical signals that drive their behavior [1,2], for example, proliferation, migration, differentiation, or ECM deposition [1–4]. Several subcellular structures, ranging from F-actin in the cytoskeleton [5] to the organization of chromatin in the nucleus [3,6], are known to participate in this process. However, a detailed understanding of the actual cellular mechanisms governing mechanotransduction remains limited. Deciphering these mechanisms will be important to develop new therapies for several diseases linked with alterations in cell mechanosensing and/or transduction [7,8], such as atherosclerosis, fibrosis, tumorigenesis, or tendinopathy [9].

In this context, the discovery of the mechanosensitive PIEZO ion channels has opened an important frontier in the field of mechanobiology and raised the possibility of exploring them as therapeutic targets [10]. These channels have been implicated in a wide variety of physiological processes, including proprioception [11] and tactile sensation [12], or cardiovascular [13,14] and musculoskeletal development [15, 16]. While PIEZO2 has been mostly associated with sensory roles in the nervous system, PIEZO1 has been studied in a broader array of tissues, including the musculoskeletal, respiratory, urinary, and vascular systems [17,18]. PIEZO1 has been shown, for example, to play a major role in tendon shear stress mechanosensing, and individuals with a gain-of-function mutation of this gene demonstrate enhanced jumping performance [19–21]. Interestingly, PIEZO1 has also been associated with various musculoskeletal disorders; for example, its deficiency in osteoblasts leads to bone loss, increased resorption, and spontaneous fractures [22], while its overexpression and excessive activity have been linked to osteoarthritis development and progression [23]. Further understanding the intricate functionality of PIEZO1 and its mediated mechanisms, particularly in musculoskeletal development and response to injury, could therefore open a path to medical breakthroughs.

PIEZO1 is an unusually large transmembrane protein composed of 2521 amino acids, assembling into homotrimers with a complex structure resembling a three-bladed propeller [24,25]. It contains 36 transmembrane helices (TM) that organize into bundles of four, called PIEZO repeats [24], which form the three flexible blade regions that extend out- and upward from the center of the homotrimer. The three PIEZO1 protomers converge to form the central ion-conducting pore structure, topped extracellularly by the C-terminal Cap domain. This unit, which is flexibly linked to the transmembrane domain, is thought to have a main role in mediating ion accessibility to the pore [24,26], being particularly crucial for channel inactivation [27]. An intracellular beam structure, positioned almost parallel to the cell membrane, links the blade periphery to the intracellular C-terminal domain at the central ion channel. Crystallographic and functional studies have suggested that force-induced conformational changes in the distal blades are transferred through the beam into the ion channel, constituting a possible lever-like gating mechanism [24,28]. However, the widely different mechanical gating kinetics of PIEZO1 in many cell types and the versatility of triggered downstream signaling responses are quite intriguing yet effectively unexplored. Current knowledge in the field suggests that the intricate 3D protein structure, as well as its interaction with various other proteins and lipids of the cell’s molecular machinery [29–33], may allow several activation routes, where different ultra-structure domains can trigger separate signaling cascades [34,35]. However, a full understanding of PIEZO1 structure-function relationships and gating mechanisms, especially in biologically relevant scenarios, remains elusive [26,28,36,37].

Studying these complex phenomena is highly challenging due to two main current experimental barriers: typical mechanical activation methods involving cell membrane indentation or stretching do not have molecular-level precision [18], while ligands targeting selected PIEZO protein domains with submolecular precision are not yet available. Therefore, new tools overcoming these barriers, allowing direct interaction with PIEZO1 in a contactless mode, would represent a major advance in the field. In this context, it has been demonstrated that mechanosensitive cell membrane receptors can be activated through remote magnetic actuation via antibody-labeled magnetic nanoparticles, with a downstream impact on cell fate [38,39]. For example, Lee and co-workers engineered a powerful magnetically-responsive nanoparticle assembly that could deliver mechanical forces specifically to PIEZO1 in mouse neurons *in vivo* via long-range magnetic stimulation [40]. This type of system can be included under the umbrella of magnetogenetics, a rising field that takes advantage of the unmatched centimeter-scale penetration depth of magnetic fields into biological tissues compared to alternative contactless stimuli, such as light [41,42].

Despite these advances, previously reported technologies face significant limitations that hinder their wide adoption as *in vitro* screening platforms and, in particular, as potential nanomedicines. For example, to focus stimulation specifically on PIEZO1, the previously mentioned technology requires transfection of target cells to express a tagged version of the channel, enabling its recognition by the nanoactuator [40]. This complex and costly approach makes it unlikely to be considered as a potential therapeutic option. Moreover, the use of antibodies against wild-type PIEZO1 is also an unreliable molecular recognition strategy, with recent studies showing that over 50 % of all commercial antibodies failed to specifically and/or sensitively recognize their target [43], an issue that has also been reported for some anti-PIEZO1 antibodies [44]. Additionally, despite recent strides in antibody discovery methodologies [45], these are typically developed against the whole protein instead of specific submolecular determinants, posing limitations to more refined structure-function studies. Other biomolecular options, such as aptamers, peptides, or affibodies, could be considered as alternatives for developing targeted ligands for selected epitopes. However, the time and resources required to generate these types of protein-binding partners would make it impractical for most laboratories [46–48]. Therefore, the development of advanced tools capable of selectively actuating PIEZO1 proteins with sub-molecular precision appears to be crucial for advancing our understanding of its structure-function relationships in cellular mechanosensing. This strategy may not only hold potential for identifying novel molecular targets, but also for being used as nanomedicines for therapeutic purposes.

**Fig. 1:**
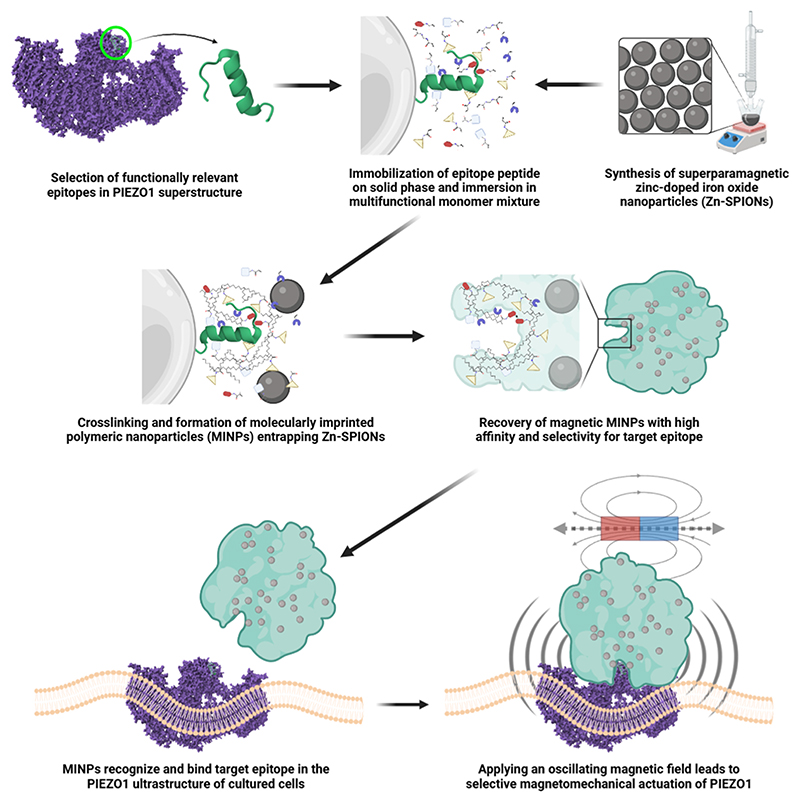
Schematic overview (not depicted to scale) of the proposed wireless actuation platform based on tailored-made abiotic nanoswitches targeting specific PIEZO1 domains (PINS).

Here, we propose a new *wireless actuation platform for interfacing with specific and potentially functionally distinct PIEZO1 domains based on tailored-made abiotic nanoswitches* (Fig. 1). These ‘PIEZO1-targeting NanoSwitches’ (PINS) consist of magnetic molecularly imprinted polymeric nanoparticles (MINPs) rationally synthesized to recognize specific epitopes on the PIEZO1 superstructure. Molecular imprinting involves the polymerization of monomers with different chemical functionalities in the presence of a template molecule. After its removal, the resulting polymer is endowed with cavities that can recognize and rebind the target molecule corresponding to the imprinted template. Since the double inception of epitope- and surface-molecular imprinting, the combination of the two techniques has provided a high level of tailored construction of “plastic antibodies” for numerous target proteins [46, 47]. Compared to biological binding partners, MINPs offer significant advantages in terms of stability, risk of eliciting immunogenic reactions, as well as speed, flexibility and cost of design and implementation [48, 49]. Importantly, MINPs offer two additional edges that enable the proposed strategy. First, they can be chemically modified or combined with other nanomaterials during synthesis to provide additional functionalities (e.g., fluorescence or magnetic responsiveness) [50,51]. Herein, superparamagnetic nanoparticles are incorporated into MINPs, allowing their remote manipulation using controlled magnetic fields to provide directed actuation capabilities. Second, the target peptide sequence can be designed a priori on demand to allow highly specific recognition of region-specific epitopes of the PIEZO1 superstructure.

We show that PINS attain high affinities for two epitopes selected from different protein domains theorized to participate in PIEZO1-mediated mechanotransduction, and that they can recognize and bind these cell receptors *in vitro*. We then demonstrate that actuation of cell-bound nanoswitches can remotely modulate PIEZO1 signaling, regulating several genes downstream of its activation. Our results show for the first time that actuation of different protein epitopes triggers widely different gene expression patterns in mesenchymal stem cell responses, including musculoskeletal differentiation programs, implying different functional roles for each domain. Overall, this approach offers an unprecedented contactless tool for studying PIEZO1 mechanosensing in appropriate biological contexts, which might ultimately contribute toward the development of innovative treatments having these receptors as therapeutic targets.

## 2 Results

### 2.1 In silico selection of biologically relevant epitopes from PIEZO1 superstructure

The first step in the synthesis of optimal MINPs was the rational selection of the imprinting epitope template. To make this selection process more efficient and rational, we followed a bioinformatic approach termed *fingerprint analysis*, consisting of *in silico* digestion of the human PIEZO1 sequence and ranking the resulting peptides for their uniqueness in the UniProtKB/SwissProt human protein databases [52]. In order to obtain a good imprinting quality and specificity for macro-molecular targets using the epitope template approach, several criteria must be considered. The first one is related to the length of the epitope peptide, which should be between 8 and 15 amino acids (a.a.) [46]. As general rules in the field, peptides shorter than 8 a.a. are considered too short to uniquely represent the epitope of a single protein, having a higher likelihood of corresponding to multiple targets. On the other hand, excessive peptide length increases the probability of generating intramolecular interactions, which can result in undesirable peptide conformations that do not correspond to the target domain, thus negatively affecting the imprinting efficiency. A second and critically important condition is that epitope templates must be derived from surface-exposed regions of the protein in its native conformation to allow its recognition and binding by the corresponding MINPs. Additionally, considering that PIEZO1 is a transmembrane protein, epitope selection must be restricted to extracellular protein regions to allow this interaction in its natural environment.

**Fig. 2:**
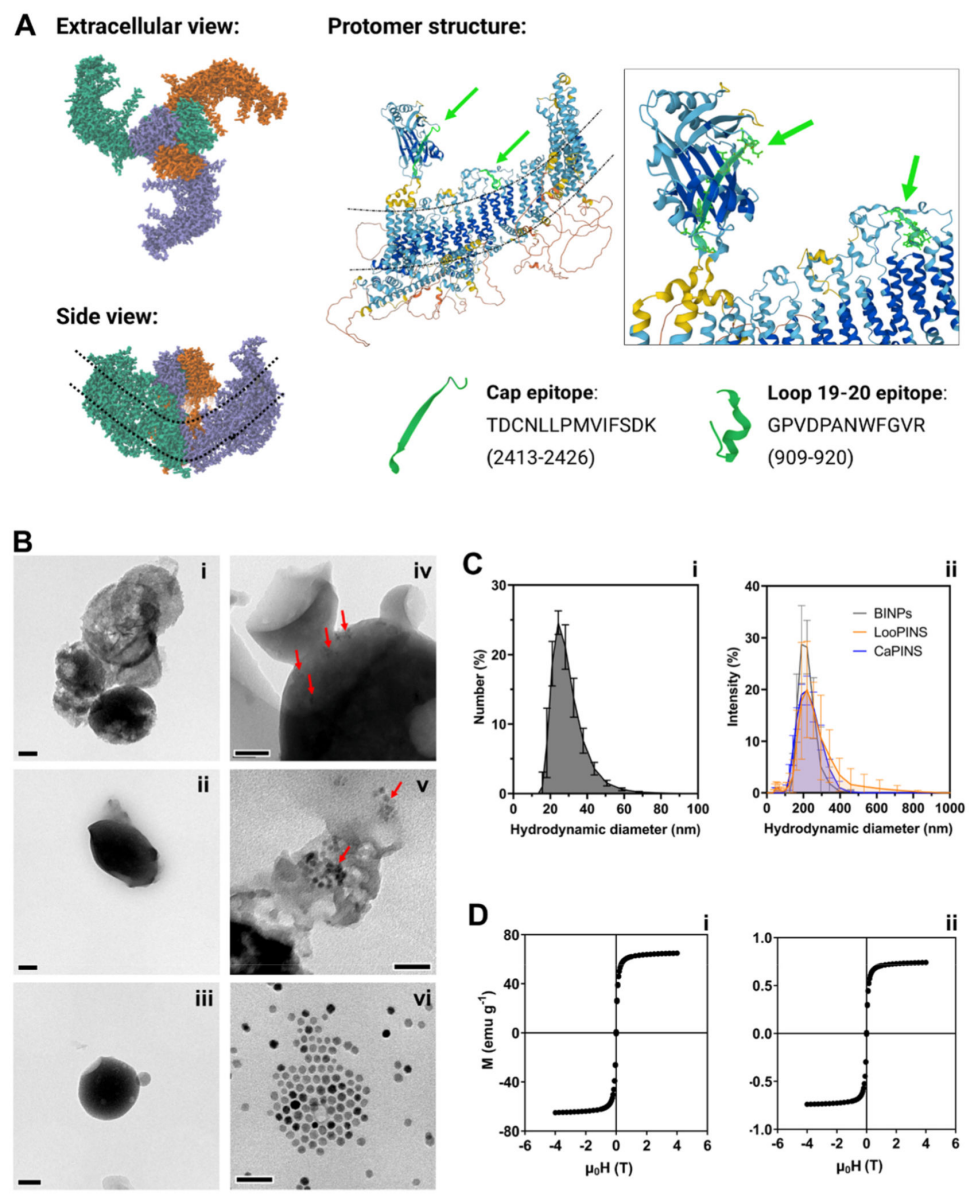
**A** - General view of the human (h)PIEZO1 trimeric protein structure and location of the two selected epitope sequences (highlighted in green) in the hPIEZO1 protomeric structure (UniProt [53] entry Q92508), corresponding to the “Cap-structure” at the C-terminal extracellular domain and to the TM19-TM20 loop region. The black dotted lines represent the approximate boundaries of the cell membrane. Adapted from AlphaFold Protein Structure Database under Creative Commons Attribution 4.0 (CC-BY 4.0) license terms [54,55]. **B-D** – Physicochemical characterization of synthesized molecularly imprinted nanoparticles (MINPs). **B** – Transmission electron microscopy (TEM) images of i) Cap epitope-imprinted PIEZO1-targeting NanoSwitches (CaPINS), ii) Loop 19-20 epitope-imprinted PINS (LooPINS), and iii) biotin-MINPs (BINPs) at 10 000× magnification. CaPINS are also shown at iv) 25 000× and v) 40 000× magnification, demonstrating the incorporation of zinc-doped superparamagnetic iron oxide nanoparticles (Zn-SPIONs – red arrows). vi) TEM image of Zn-SPIONs at 40 000× magnification (scale bars: 100 nm). **C** – Dynamic light scattering analysis of size distribution of i) Zn-SPIONs and ii) MINPs. Data presented as mean ± standard deviation (SD). Statistical analysis by one-way Analysis of Variance (ANOVA), followed by Tukey’s *t*-test for individual comparisons between BINPs, LooPINS, and CaPINS, showed no significant differences between the average sizes of different MINP types (n = 5 batches). **D** – Magnetometry measurements at 300 K of i) Zn-SPIONs and ii) MINPs, confirming the superparamagnetic behavior of Zn-SPIONs and their capability to provide MINPs with magnetic response.

Based on the results of this *fingerprint analysis* and the established selection criteria (Table S1, Supporting Information), we excluded all peptides that belonged to annotated intramembrane or intracellular regions, more specifically, the TM helix and intracellular beam domains. Then, we assessed the location of remaining sequences within the three-dimensional structure of the human PIEZO1 protomer, starting from the highest-scoring. Out of these, we found that the epitopes ranked 3rd, 6th, 7th, 9th, and 10th corresponded to other intracellular/intramembrane regions of PIEZO1, as shown in Fig. S1 (Supporting Information), and were therefore also excluded. By contrast, the 4th and 5th placed peptides corresponded to closely located segments in the extracellular Cap domain. Both of these sequences were found to be surface-exposed on the Cap ultrastructure when the homotrimer is assembled. This provides an interesting targeting option, since the Cap domain has been implicated in regulating ion accessibility and inactivation kinetics [27]. Given their similar overall molecular characteristics, the best scorer was selected as the template. This was a 14-mer peptide between a.a. 2413-2426 with the sequence TDCNLLPMVIFSDK, which is hereafter referred to as the ‘Cap epitope’. Fig. 2 A shows its location (highlighted in green, plus arrow) within the PIEZO1 protomer 3D structure.

From the peptide ranking table, we searched for a second epitope that would represent a different functional region of PIEZO1, ideally belonging to the blade domains. These undergo force-induced conformational changes that are transmitted via the beam domain toward the pore, probably responding to changes in membrane tension, thus representing potential differences in functionality compared to the Cap [28]. The 11th-ranked sequence (a.a. 909-920, sequence: GPVDPANWFGVR) corresponding to an extracellular loop between TM19 and TM20 (Loop 19-20) in the blade structure, matched these requirements (Fig. 2 A, green arrow and structure highlight in the inset). Importantly, this loop structure has been previously reported to have an active role in PIEZO1 mechanical activation [25,56], making it an attractive target for our actuation approach. This epitope was therefore selected as a second molecular imprinting template, henceforth named the ‘Loop 19-20 epitope’. The two custom-synthesized peptides corresponding to these sequences were then used as templates for the imprinting of two types of polymeric nanoparticles targeting these different domains in the PIEZO1 superstructure: Cap epitope-imprinted PINS (CaPINS) and Loop 19-20 epitope-imprinted PINS (LooPINS). These two target regions therefore appear as mechanically and functionally distinct within the PIEZO1 architecture, providing a robust basis to explore the proposed strategy and its downstream biological implications.

### 2.2 SPIONs grant superparamagnetic behavior to MINPs

To turn polymeric MINPs into the proposed wireless nanoswitches, the incorporation of functional elements responsive to external stimuli within their structure is required. For this purpose, we decided to modify MINPs with superparamagnetic iron oxide-based nanoparticles (SPIONs), thus enabling their remote actuation through the application of external magnetic fields. When the dimensions of iron oxide-based nanostructures fall below a critical value, they enter the so-called superparamagnetic regime [57]. In this state, SPIONs exhibit a negligible remanent magnetization following exposure to a magnetic field, preventing aggregation and granting higher control over their guidance [58]. However, incorporation of SPIONs into the polymeric matrix of nanosized MINPs has a physical threshold beyond which it starts affecting the nanoparticle structure, particularly the imprinted cavity that binds the target molecule. In turn, this also constrains the potential magnetic response and corresponding actuation output of the nano-switches. To minimize these limitations while maximizing MINP actuation performance, it is crucial to design SPIONs with the highest possible magnetic power, providing MINPs with the desired magnetic response by incorporating a minimal amount of magnetic material. Here, the magnetization of the nanostructures was increased through a doping strategy, consisting of the partial substitution of iron by zinc in the magnetite-type structure of the synthesized SPIONs (Zn-SPIONs). We have previously shown that this is an efficient synthetic approach to maximize the saturation magnetization of iron oxide-based nanoparticles while preserving their superparamagnetism [57,59].

Following the synthesis of magnetic nanoparticles, three types of MINPs were produced using the solid phase imprinting method [60,61]. Besides CaPINS (Fig. 2 Bi) and LooPINS (Fig. 2 B ii), biotin-imprinted nanoparticles (BINPs, Fig. 2 B iii) were prepared as negative controls. Because this method involves the immobilization of the template molecule onto a solid phase support, it has the advantage of allowing i) the reuse of the same template in multiple syntheses, thus reducing the associated costs, and ii) the recovery of a purified high-affinity MINP fraction, since lower-affinity particles are washed out in an intermediate step. However, this implies that true non-imprinted nanoparticles cannot be synthesized. Thus, BINPs are commonly employed as appropriate negative controls in this framework. BINPs share identical physicochemical properties with PIEZO1-imprinted nanoswitches (PINS), including size, morphology, surface chemistry, and charge, but lack affinity for the PIEZO1 epitope and mechanotransduction-related targets. Throughout the manuscript, BINPs were therefore systematically used as the functional equivalent of non-imprinted nanoparticles to assess specificity.

Transmission electron microscopy (TEM) visualization of MINPs (Fig. 2 Bi-v) shows the successful synthesis of approximately spherical composite nanoparticles incorporating Zn-SPIONs (Fig. 2 B iv and B.v, red arrows), regardless of the epitope template used. Free Zn-SPIONs are shown in Fig. 2 B vi for comparison. Dynamic light scattering analysis (Fig. 2 C and Table S2, Supporting Information) globally corroborates TEM observations, confirming the successful synthesis of a highly monodisperse population of Zn-SPIONs with a hydrodynamic particle diameter of 27.7 ± 1.5 nm (average inorganic core diameter of 8.6 ± 1.9 nm, as determined from TEM images). The difference in sizes between the two techniques is attributed to the polymeric layer used for coating SPIONs in order to transfer them to aqueous media, and to the corresponding stretching of hydrophilic polymer chains when the particles are in suspension. Moreover, we could also confirm that MINPs have similar hydrodynamic sizes irrespective of the imprinted template, varying between 213 ± 18 nm for BINPs and 248 ± 37 nm for LooPINS. Polydispersity index was significantly lower for CaPINS, compared to LooPINS and BINPs, although this parameter was relatively high for the three groups. This is not surprising considering that nanoparticles are synthesized by typical free radical polymerization without additional methods for controlling polymer growth. Zeta potential measurements (Table S2, Supporting Information) show that all particles have a similar negative surface charge, which is preferable for the intended function because it will contribute to minimizing unspecific adsorption of nanoparticles to the cell membrane.

As previously discussed, a critical requirement for the functionality of the proposed nanoswitches is magnetic responsiveness. Therefore, we confirmed that Zn-SPION incorporation endowed MINPs with super-paramagnetic behavior, as seen by the negligible hysteresis loop in CaPINS magnetometry measurements (Fig. 2 D ii), nicely replicating free Zn-SPION curves (Fig. 2 Di). Regarding the values of saturation magnetization (Table S3, Supporting Information), it is possible to calculate that the value for CaPINS (0.740 emu g^−1^) was 1.14 % of the value for pure Zn-SPIONs (65.0 emu g^−1^). Considering that the initial mass of Zn-SPIONs represented 1.22 wt% of the total monomer mixture before polymerization and assuming that the polymeric matrix does not significantly alter their magnetic responsiveness, we can conclude that Zn-SPIONs were incorporated into MINPs almost in direct proportion to their starting mass fraction. Overall, these results show that the developed strategy allowed the effective synthesis of magnetically responsive nanoswitches for different target epitopes without major differences in their essential physicochemical properties.

### 2.3 Nanoswitches recognize and bind PIEZO1 with very high affinity

**Fig. 3:**
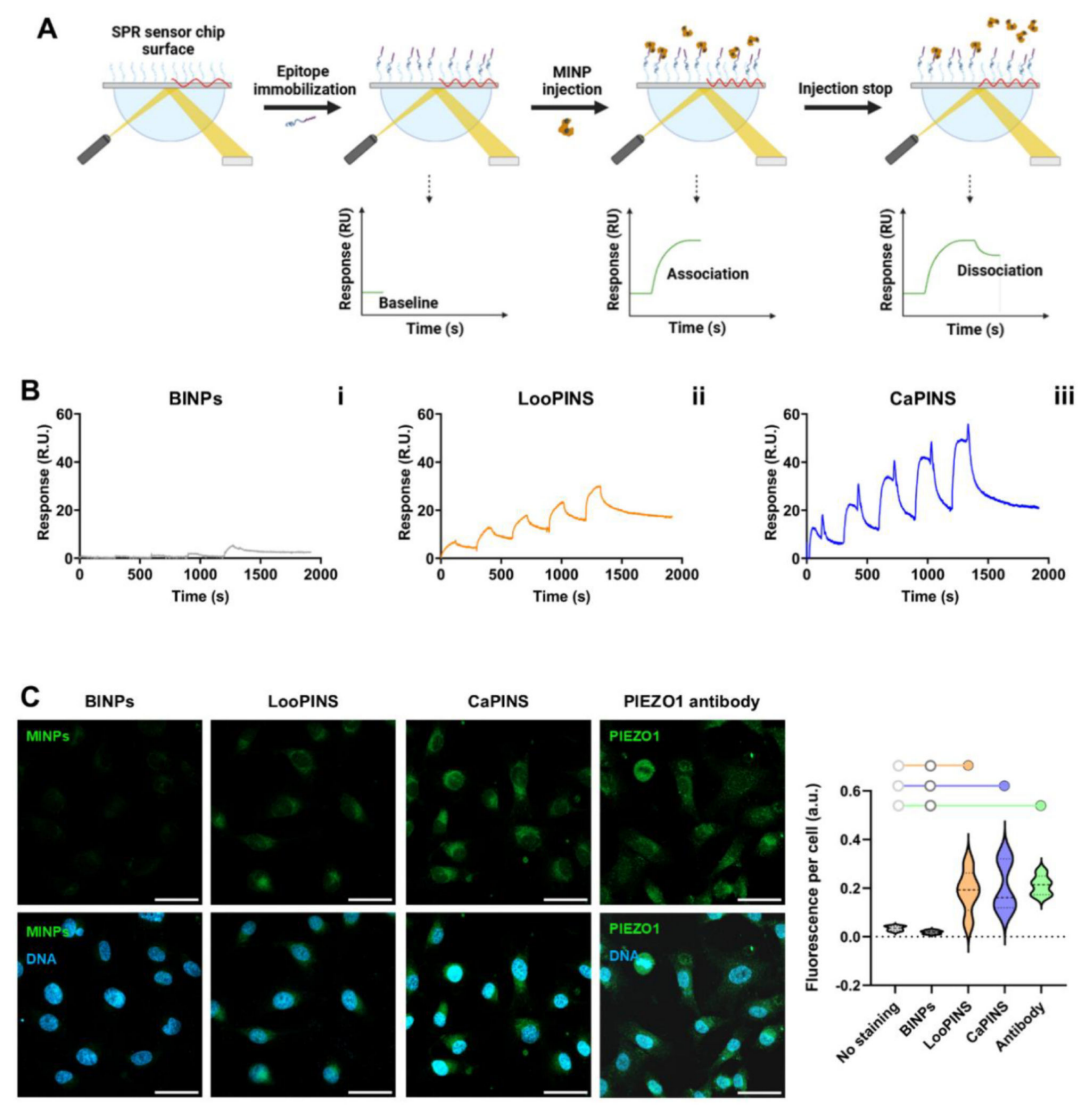
Assessment of PIEZO1-targeting NanoSwitches (PINS) rebinding to the PIEZO1 channel. **A** – Scheme of a typical SPR kinetic assay and corresponding readings of each step. **B** – representative sensorgrams for i) biotin-imprinted nanoparticles (BINPs, grey) binding to PIEZO1 Loop 19-20 epitope; ii) Loop 19-20 epitope-imprinted PINS (LooPINS, orange) and iii) Cap epitope-imprinted PINS (CaPINS, blue) binding to their respective target epitopes. Further details regarding sensorgrams are included in Table 1 and S3 (Supporting Information). **C** - Fluorescence microscopy visualization of MINP recognition and binding to PIEZO1 in cultured EA.hy926 endothelial cells (scale bars: 50 μm). Green – fluorescein-modified MINPs/anti-PIEZO1 antibody; blue – DNA (4,6-diamidino-2-phenylindole staining). Box plot represents quantification of green fluorescence levels normalized by the number of nuclei present in each image. Data presented as violin plots showing the distribution and density of the data, with the internal marks indicating the median and interquartile range (n = 5 images per condition; n = 3 biological replicates). Statistical analysis performed by one-way Analysis of Variance (ANOVA), followed by Tukey’s *t*-test for individual comparisons (α = 0.05). Fully colored circles indicate that the respective experimental group is significantly different (p < 0.05) from groups identified by white circles.

Next, we assessed if the imprinting procedure could generate significant affinity of PINS for the target epitope template using surface plasmon resonance (SPR). Fig. 3 A schematizes a typical SPR kinetic assay and the corresponding expected outcome. Since MINPs prepared by solid phase imprinting are likely to present only a few binding cavities per nanoparticle, their immobilization onto the SPR sensor chip could lead to the masking of the imprinted sites and thus block peptide rebinding. To minimize such effects, we opted instead for covalently immobilizing the epitope peptides on the sensor chips through carbodiimide chemistry, linking lysine residues to carboxyl groups present on the chip surface. Thus, epitopes were used as the stationary ‘ligands’ for these assays, while nanoswitches were injected in suspension as the ‘analyte’. We started by challenging a sensor functionalized with the PIEZO1 Loop 19-20 epitope with BINP suspensions at increasing concentrations. BINPs were selected as control nanoparticles since their template molecule is completely unrelated to either PIEZO1 epitope, but they still retain similar chemical nature, size and surface charge distributions compared to epitope-imprinted nanoparticles, essentially functioning like non-imprinted nanoparticles [62]. As can be seen in Fig. 3 Bi, signal response to BINP injections is very low throughout the analyzed range of concentrations. Thus, fitting these curves to a 1:1 binding model did not allow the calculation of kinetic rate constants, with U-values of 43-95 (a.u.), far beyond the acceptable limit (as reference, U-values above 25 indicate that absolute values for two or more of the parameters could not be determined independently).

In contrast, a marked stepwise increasing response was observed when injecting PINS against each respective target epitope, showing that both LooPINS (Fig. 3 B ii) and CaPINS (Fig. 3 B iii) generated clearly defined association/dissociation curves when interacting with their corresponding template peptide. The standard 1:1 binding model provided good quality fits for both types of PINS, closely describing the resulting sensorgrams. In both cases, there is a significant contribution from bulk effects in the observed response, which is expected given the large size of MINPs in comparison to proteins and other smaller molecules typically analyzed in SPR. After subtracting these effects, we could isolate the specific binding component in each fitting, thereby allowing the independent calculation of kinetic rates and, subsequently, the corresponding equilibrium dissociation constant (*K*_*D*_), as summarized in Table 1 (further detail in Table S4, Supporting Information). Association rates were quite similar between the two epitopes, comparing favorably with some of the highest *k*_*a*_ values reported for monoclonal antibodies [63]. LooPINS presented a relatively lower (*approx*. 2.8-fold) dissociation rate, indicating a more stable interaction with the target peptide compared to CaPINS. Nevertheless, both nanoswitches displayed *K*_*D*_ in the sub-nanomolar range, falling within typical values of commercially available monoclonal antibodies [63], and being among some of the highest affinity constants for synthetic MINPs developed against proteins reported so far [46]. These results demonstrate that the imprinting process was effective in generating specific affinity of the magnetically-responsive nanoswitches for both target epitopes.

After confirming that nanoswitches showed a remarkable affinity for their target epitope, we investigated whether this ability translated to the recognition of the full PIEZO1 protein in cultured cells. To that end, green fluorescent versions of MINPs were synthesized by incorporation of fluorescein-O-methacrylate in the polymerization mixture, allowing their visualization by fluorescence microscopy (Fig. S2, Supporting Information), and used as ‘synthetic antibodies’ for immunolabeling of the target protein [64]. MINPs were incubated with *in vitro* cultured endothelial cells (EC; EA.hy926 cell line), in analogy to how antibodies are usually applied in immunocytochemistry procedures. ECs were chosen here as reporter cells for this assay since they are well-known to highly express PIEZO1. In fact, PIEZO1 is a key sensor of the shear stress associated with blood circulation, being chiefly responsible for EC homeostasis and adaptation to changes in blood flow conditions [65,66]. As shown in Fig. 3 C, no significant fluorescence signal could be detected in ECs incubated with BINPs, similar to the images obtained for the group without MINPs. In contrast, PINS appeared to recognize and bind cells in a stable manner. Fluorescence intensity in CaPINS and LooPINS groups was significantly higher than both negative controls (p < 0.01), and comparable to the labeling obtained with anti-PIEZO1 antibody (no significant differences between these three groups). Globally, these results indicate that nanoswitches for both PIEZO1 epitopes can not only recognize and bind their target peptide, but also the corresponding parent full protein in its native conformation on cell membrane surfaces.

### 2.4 Magnetic stimulation through nanoswitches triggers PIEZO1 activity

Having shown that synthesized magnetically responsive MINPs can recognize and bind specific PIEZO1 epitopes, next we sought to assess their potential to actuate these receptors and what downstream signaling effects they elicit according to the targeted domain. Before directly probing the nanoswitches in live cells, we performed *in silico* simulations for the magnetic systems to be used (Figs. S3 and S4, Supporting Information), estimating the magnetic forces that PINS can apply to the PIEZO1 protein via torque. The resulting values lie in the range of 83-400 pN. Previous studies have measured the minimum force required to shift the PIEZO1 blade conformation in the order of dozens of piconewton [26,37], meaning that these nanoswitches are likely able to elicit a robust gating of PIEZO1.

**Fig. 4:**
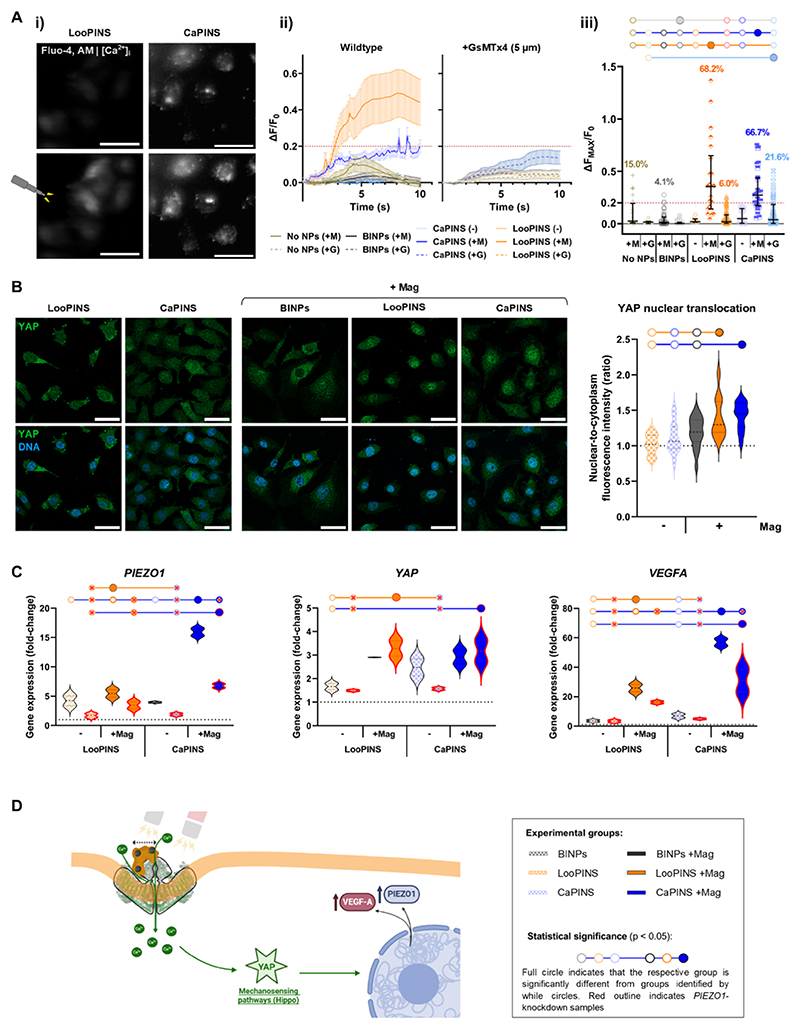
Assessment of PIEZO1 gating by remote magnetic actuation via nanoswitches in EA.hy926 endothelial cells (ECs). **A** – Live intracellular calcium ([Ca^2+^]_i_) imaging during actuation of nanoswitches via approximation of a magnetic probe. **i)** Total Internal Reflection Fluorescence (TIRF) microscopy images showing ECs incubated with nanoswitches in the moment before (up) and 4 s after (down) stimulation with the magnetic tip. **ii)** Quantification of fluorescence levels (corresponding to [Ca^2+^]_i_) per cell from TIRF microscopy images during a 10 s time window encompassing stimulation via magnetic tip (+M; dark continuous lines). ECs incubated with GsMTx4 (5 μm) calcium ion channel inhibitor (+G; dashed lines) or without magnetic stimulation (minus symbol [–]; light continuous lines) were used as negative controls. Results expressed as proportional variation (ΔF) over the baseline (F_0_, *t* = 0). An increase of 20 % (0.2, dotted line) was considered the threshold for PIEZO1 activation in each cell [67]. A minimum of three fields of view (FOV), in two biological replicates, for a total of >17 cells, were acquired for each experimental group. The same FOV was used for stimulated vs. unstimulated samples, to maintain consistency. Data presented as mean ± standard error of mean (SEM). **iii**) Distribution of the maximum fluorescence intensity per cell measured in TIRF microscopy images (ΔF_MAX_). The proportion of cells in each group that surpassed the 20 % activation threshold are shown as percentages in the plot. Data presented as median ± interquartile range (IQR) along with individual values. Statistical analysis performed by Kruskal-Wallis test, followed by Dunn’s test for individual comparisons (α = 0.05) after removal of outliers by Robust regression and Outlier removal (ROUT) method (Q = 1%). Fully colored circles indicate that the respective experimental group is significantly different (p < 0.05) from groups identified by white circles. Full statistical analysis in Table S5 (Supporting Information). **B** – Immunocytochemistry staining of YAP (Yes1 associated transcriptional regulator) intracellular localization in cells under (+Mag) or without the application of an external shifting magnetic field (scale bars: 50 μm). Green – anti-YAP antibody; blue – DNA (4,6-diamidino-2-phenylindole staining). Violin plot represents the quantification of nuclear-to-cytoplasmic ratio of green fluorescence levels (biological replicates: n = 3; number of images acquired per group was >13). The dotted line indicates a ratio of 1 – equal distribution between cytoplasm and nucleus. Data presented as violin plots showing the distribution and density of the data, with the internal marks indicating the median and IQR. Statistical analysis performed by one-way analysis of variance (ANOVA), followed by Tukey’s *t*-test for individual comparisons (α = 0.05). Complete data for statistical analysis are displayed in Table S6 (Supporting Information). **C** – Expression of select genes downstream of PIEZO1 activity in ECs after 7 days of culture. The red outline indicates *PIEZO1-KD* groups. Quantitative real-time polymerase chain reaction (qPCR) results are normalized to transcript levels of *GAPDH* (glyceraldehyde 3-phosphate dehydrogenase) and displayed as fold changes relative to gene expression in day 0 (incubation with nanoswitches) (n = 3 biological replicates; 2 technical replicates). Data presented as violin plots showing the distribution and density of the data, with the internal marks indicating the median and IQR. Statistical analysis by two-way ANOVA followed by Tukey’s post hoc tests for individual comparisons between experimental groups (α = 0.05). The dotted line represents the basal expression for each marker (“1-fold”) on experimental day 0. Complete data for statistical analysis are displayed in Table S8 (Supporting Information). *PIEZO1* – Piezo Type Mechanosensitive Ion Channel Component 1; *YAP* – Yes1 associated transcriptional regulator; *VEGFA* – vascular endothelial growth factor A. **D** – Schematic depiction of the main findings in ECs. Actuation of the PIEZO1 Cap region leads to YAP nuclear translocation, upregulating *PIEZO1* and *VEGFA* in a PIEZO1-dependent manner.

Next, to directly assess functional PIEZO1 activation in real time, intracellular calcium dynamics were monitored using live-cell total internal reflection fluorescence (TIRF) microscopy, to preferentially probe calcium signals arising at or near the plasma membrane, where PIEZO1 channels are localized. Magnetic stimulation was applied using a custom magnetic tip composed of a vertically stacked array of permanent magnets, positioned in close proximity to the cell monolayer to generate a localized magnetic field gradient (Fig. S3, Supporting Information). This configuration enabled controlled actuation of surface-bound nanoswitches while remaining fully compatible with live imaging. Changes in the fluorescence intensity of the Fluo-4 calcium-sensitive dye were recorded before, during, and after magnetic stimulation, allowing quantitative assessment of calcium influx in response to nanoswitch actuation (Fig. 4 A and Videos 1–3). Results showed pronounced and sustained increases in [Ca^2+^]_i_ after stimulation of cells incubated with CaPINS and LooPINS (Fig. 4 A i and ii, Videos 1 and 2). Interestingly, a transient (~5 s) calcium response of lower magnitude could also be seen in cells with no nanoparticles (NPs) or with BINPs when under magnetic stimulation (Video 3 and Fig. 4 A ii). This suggests some inherent sensitivity of cells to magnetic fields may be leading to increased [Ca^2+^]_i_ influx, which is consistent with what is known for other ion channels, although other non-specific indirect effects from the probe-based actuation cannot be ruled out. Nevertheless, the maximum response level was significantly lower than for cells stimulated with PINS and was not sustained over time. Moreover, the proportion of activated cells – defined as surpassing a 20 % increase above baseline [Ca^2+^]_i_ – in the No NPs and BINPs groups was significantly lower (15.0 % and 4.1 %, respectively) compared to both CaPINS- (66.7 %) and LooPINS- (68.2 %) stimulated groups, both of which showed remarkable responses (Fig. 4 A iii). Crucially, the response of cells incubated with BINPs was not significantly different from those with no NPs, thereby indicating that the response observed in both PINS groups was specifically due to PIEZO1 actuation via bound nanoswitches.

Supplementary data related to this article can be found online at https://doi.org/10.1016/j.bioactmat.2026.03.051

To further confirm this specificity, we repeated this assay in cells treated with GsMTx4 (Grammostola mechanotoxin 4), a spider venom peptide that inhibits cationic mechanosensitive channels including PIEZOs [10]. Despite not being a specific PIEZO inhibitor and not directly acting on the channel itself, GsMTx4 acts by inserting into the cell membrane, modulating local membrane tension. This increases the gating threshold of ion channels and thus lowers their responsiveness to mechanical stimuli. Indeed, GsMTx4 treatment drastically decreased Ca^2+^ signaling in all experimental groups, including nearly abolishing response to stimulation by LooPINS (Fig. 4 A ii & iii). Interestingly, although it significantly dampened the effect of CaPINS actuation (66.7 % to 21.6 %, p < 0.0001), its effect on this group was less pronounced than that observed for LooPINS (68.2 % to 6.0 %, p < 0.0001), with significant Ca^2+^ responses to CaPINS still being present in GsMTx4-treated cells. This suggests different mechanistic roles for each domain in PIEZO1 channel gating. Considering the mode of action of GsMTx4 and the 3D structure of PIEZO1, it is plausible that the Cap region is less sensitive to the effects induced by GsMTx4 on the cell membrane, thus allowing a partial bypass of the toxin-induced inhibition in this assay.

Next, to analyze biological responses downstream from PIEZO1 gating, which happen in a longer time frame, a different magneto-mechanical actuation setup was used (Figure S4 A & B, Supporting Information). ECs were first incubated with each type of MINP and cultured for up to 7 days in the presence (+Mag) or in the absence (–) of cyclical magnetomechanical stimulation. To actuate the nanoswitches, we used a well-established magnetic system that has been previously applied in several other *in vitro* cell magnetomechanical stimulation settings [68], consisting of an array of permanent magnets placed under the cell culture well plates, with each magnet aligning with an individual well. A horizontal magnet shift of 0.2 mm at a moderate movement frequency of 2 Hz was applied, with an average magnetic field (|*B*|) delivered at each cell position of 467 ± 56 mT, according to the Finite Element Method Magnetics (FEMM) simulation (Figure S4 C & D, Supporting Information) [68].

After confirming that MINPs did not negatively affect cell viability during this culture period (Fig. S5, Supporting Information), we explored possible mechanotransduction effects downstream of PIEZO1 activity. Among these, we focused on Yes1-associated transcriptional regulator (YAP), a mechanosensitive transcriptional coactivator (alongside transcriptional co-activator with PDZ-binding motif – TAZ) responsible for integrating biophysical signals into differential gene expression [2,69,70]. A growing body of evidence increasingly shows that YAP is directly linked to PIEZO1 activity, possibly by working in a mutual feedback loop [71–73]. To that end, we analyzed the subcellular localization of this protein by immunofluorescence. YAP is primarily located in the cytoplasm, shuttling to the nucleus upon cellular mechanotransduction activation [68]. Indeed, remote actuation via either LooPINS or CaPINS caused significant increases (p < 0.01) in nuclear YAP fluorescence when compared with non-stimulated samples (nuclear-to-cytoplasmic ratios of 1.4 ± 0.3 vs 1.0 ± 0.1 for LooPINS and 1.4 ± 0.2 vs. 1.1 ± 0.2 for CaPINS) (Fig. 4 B). It is relevant to note that the magnetic field alone has been reported to have some influence over YAP activity [74], which could be related to the transient effect observed for magnetic field gating of PIEZO1 without PINS. Although this effect can also be seen to some degree for the BINP + Mag group, its nuclear-to-cytoplasmic ratio (1.2 ± 0.2) was not significantly different from the non-stimulated samples, while being significantly lower than for samples actuated on either PIEZO1 epitope (p < 0.05). These results support the hypothesis that magnetic actuation of PINS can specifically transfer a mechanical input onto PIEZO1 channels, causing the activation of mechanotransduction pathways.

Next, we sought to confirm if this increase in YAP activity also corresponded to changes in cell transcription responses by analyzing the expression of gene markers that have been associated with immediate downstream effects of PIEZO1 activation, namely *YAP* and *PIEZO1* themselves (Fig. S6, Supporting Information) [71,75,76]. Remarkably, magnetic actuation of PIEZO1 Cap upregulated expression levels of both genes, which were significantly higher than in control (BINPs) and LooPINS groups (*approx*. 2.3-fold for *YAP* and 2.9-fold for *PIEZO1*). Interestingly, we also observed an upregulation trend in CaPINS groups in the absence of the magnetic field, which was statistically significant for *PIEZO1*, suggesting that the mere binding of nanoswitches to the Cap region has a cell signaling effect. To bolster these results, we looked for further downstream response markers for PIEZO1 activity. One of the most well-studied roles of PIEZO1 is in the development and adaptation of the vascular system [77], in particular, in the angiogenesis process [78,79]. Thus, we looked for one of the most prolific pro-angiogenic growth factors, vascular endothelial growth factor A (VEGF-A) [80]. The results are particularly consistent with those of *YAP* and *PIEZO1*, showing *VEGFA* upregulation by CaPINS actuation. Globally, the resulting gene expression patterns observed for these three markers support the hypothesis that remote actuation of PIEZO1 Cap might promote a pro-angiogenic-like phenotype in cultured ECs, consistent with the known signaling role of this receptor in native tissues. In fact, *VEGFA* is known to be upregulated by increased shear, most likely via PIEZO1 mechanosensing and transduction pathways [77–79].

To unequivocally understand if these observed transcriptional responses derived specifically from PIEZO1-dependent signaling, we repeated the oscillating magnetic actuation setup using cells transfected with an siRNA targeting PIEZO1, resulting in a knockdown of PIEZO1 expression (*PIEZO1-KD*) for up to four days of the 7-day culture period. Real-time quantitative polymerase chain reaction (qPCR) results (Fig. 4 C) show a consistent trend of lower expression for both *PIEZO1* and *VEGFA* in *PIEZO1-KD* cells. Wild-type cells magnetically actuated by CaPINS show the highest upregulation for both markers, consistent with the day 1 results (Fig. S6, Supporting Information), but also the most pronounced decrease in the *PIEZO1-KD* group. Although this decrease did not reach the basal level, this was expected considering that the silencing effects only lasted during a part of the culture period. Therefore, PIEZO1 activity seems to be directly linked to the regulation of these two genes through increased nuclear translocation of YAP, particularly when the Cap domain is involved. Interestingly, the transcriptional profile of *YAP* did not follow a similar pattern, apparently correlating with the application of the magnetic field itself, but not with either type of PINS. *PIEZO1*-*KD* groups actually showed a statistically significant increase in *YAP* level compared to the baseline for this marker. Nevertheless, these were very close to the PIEZO1 wild-type groups, indicating a lack of influence by PIEZO signaling in the regulation of *YAP* transcription at this timepoint.

Altogether, this initial functional analysis indicates that magneto-mechanical actuation of tailor-made PINS effectively modulates PIEZO1 activity (Fig. 4 D). Furthermore, we could also see different response patterns according to the targeted epitope. According to these results, it is reasonable to hypothesize that PIEZO epitopes might have different functional roles, each one participating in alternate mechanosensing mechanisms, potentially triggering distinct signaling pathways and/or requiring other partners to exert specific effects (e.g., PIEZO1-binding proteins [31], extracellular matrix [67]). These can, in turn, lead to different cellular responses, of which we only analyzed a few markers.

### 2.5 Targeted submolecular PIEZO1 actuation modulates mesenchymal stem cell phenotype in an epitope-dependent manner

After confirming the capability of PINS to actuate on selected PIEZO1 domains, we next explored this technology to probe whether downstream effects of region-specific PIEZO1 stimulation may differentially affect stem cell signaling and fate determination. Envisaging a potential application in regenerative therapies for musculoskeletal diseases, human adipose tissue-derived stem cells (hASCs) were used for these studies. hASCs are a common mesenchymal stem cell (MSC) source with self-renewal and multipotency capacity that can be differentiated into several musculoskeletal cell types (e.g., osteoblasts, chondrocytes, myocytes, tenocytes), having the additional advantage of being more widely available and easier to harvest than other sources, such as bone marrow [81,82]. For these assays, hASCs were first incubated with each type of nanoswitch and then subjected to the magnetomechanical stimulation protocol previously described for ECs. Cells treated with BINPs and the same magnetomechanical stimulation were used as the control group. After one week, the PIEZO1 actuation effects on cell transcription responses were analyzed by bulk RNA sequencing (RNA-seq).

**Fig. 5:**
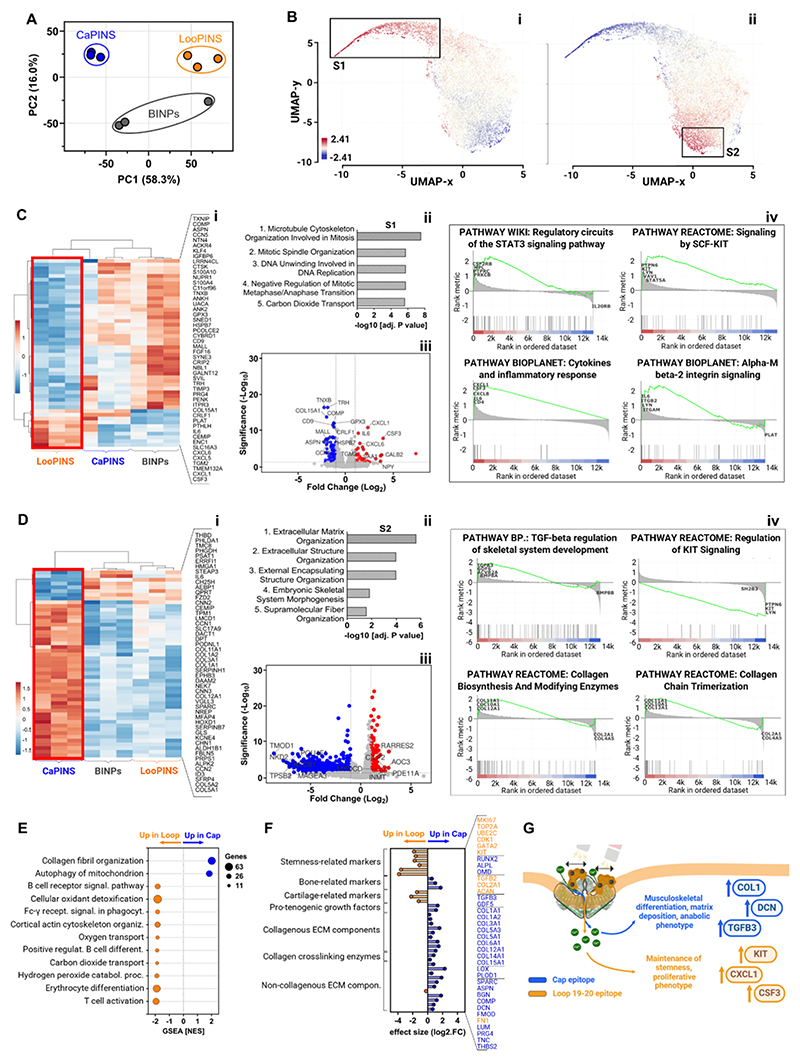
Transcriptomic analysis of human adipose tissue-derived stem cells (hASCs) under magnetomechanical actuation through PIEZO1-targeting NanoSwitches (PINS). **A** – Principal component (PC) analysis of experimental groups (BINPs – biotin-imprinted nanoparticles; CaPINS –Cap epitope-imprinted PINS; LooPINS –Loop 19-20 epitope-imprinted PINS) performed on the expression data for the top 6711 differentially expressed genes. **B** - Gene signature maps for (i) LooPINS and (ii) CaPINS groups. Graphs show UMAP (Uniform Manifold Approximation and Projection for Dimension Reduction) clustering of genes colored by differential log-expression relative to the average of BINP groups. The distance metric is covariance; genes that are clustered nearby have high covariance. **C**,**D** – Global transcriptomic profiling of (C) LooPINS groups and (D) CaPINS groups compared to control (BINP) groups. **i**) Heatmap of gene-level hierarchical clustering of the top 50 differentially expressed genes (DEG). Columns represent individual samples (n = 3 biological replicates). Blue denotes downregulated genes; orange denotes upregulated genes. **ii**) Top 5 gene ontology (GO) annotations of S1 (C.ii) and S2 (D.ii) subsets of genes in UMAP (B), corresponding to the most upregulated gene clusters in LooPINS and CaPINS groups, respectively. Performed using *Enrichr*, queried against the GO Biological Process 2023 database. Bar plot represents -log_10_[adjusted P value] for the Fisher-weighted, average correlations of the subset with the annotation terms in the queried database. **iii**) Volcano plot of DEGs. Colored dots show the 96 (C.iii) or 678 (D.iii) significantly expressed genes, as determined by *kallisto*, with the horizontal line corresponding to a false discovery rate (FDR) < 0.05 and vertical lines are at a cutoff of log_2_[Fold change] ± 1. **iv**) Gene Set Enrichment Analysis (GSEA) enrichment plots of some of the top differentially expressed gene sets. The plot’s black vertical lines represent the ranked gene hits in the gene set. The green line represents the normalized enrichment score (NES). The more the NES curve is skewed to the upper left of the plot, the more the gene set is enriched in the corresponding experimental group. The red-to-blue colored scale at the bottom represents the degree of correlation of genes in the experimental group with the phenotype in the gene set (Red: positive correlation; blue: negative correlation). **E** – GSEA of the 2 positively enriched and the 10 most negatively enriched biological processes by NES in CaPINS groups when directly compared to LooPINS groups (adjusted p-value <0.05). The further right, the more the gene set is enriched by Cap epitope actuation; the further left, the more it is enriched by Loop 19-20 epitope actuation. **F** – Differential expression analysis between CaPINS and LooPINS groups of a pre-selection of genes related to stemness, musculoskeletal differentiation pathways (bone, cartilage, tendon), extracellular matrix (ECM) components, and collagen crosslinking enzymes. Only significantly different genes were included (p < 0.05). **G** – Schematic depiction of the main findings of transcriptomic analysis in hASCs. Actuation of each epitope leads to contrasting cell responses, suggesting different functional roles for each domain in PIEZO1-mediated mechanotransduction.

Before assessing the treatment effects on nanoswitch-stimulated experimental groups, we first analyzed whether the potential residual BINP binding to cell surfaces or the magnetic field by itself could induce any significant response on cellular phenotype, as has been recently described for MSCs [83,84]. As shown in Fig. S7 (Supporting Information), despite some differences between groups that did (+Mag) or did not undergo magnetomechanical actuation, only 31 genes were found to be differentially expressed, with a single Gene Ontology (GO) annotation significantly enriched (GO:0032103 ‘Positive regulation of response to external stimulus’). Considering the negligible unspecific BINP binding to the cell surface (Fig. 3C) and the transient influence that magnetic stimulation showed on PIEZO1 gating, this was an expected result. After excluding any major impact derived from the control group (BINPs + Mag) on cell response, we next performed a principal component analysis (PCA) to understand how the overall phenotypes of samples treated with the different nanoparticles and subjected to magnetic stimuli relate to one another. Unlike the BINP group, samples treated with the different PINS clustered closely together, forming two nearly equidistant clusters (Fig. 5 A). In line with the previous qPCR findings, we can see that samples under targeted actuation of PIEZO1 presented a clearly different phenotype from the negative control and, quite remarkably, that CaPINS and LooPINS groups also differed substantially in their transcription signature.

Delving further into the results, we mapped the gene signature of each group by performing a UMAP (Uniform Manifold Approximation and Projection for Dimension Reduction) rendering of all detected genes by covariance, using BINP samples as the reference group (Fig. 5 B). This type of visualization allows us to more precisely dissect how cell behavior diverges between the two experimental groups. Interestingly, the differential gene expression responses induced by actuating PIEZO1 through the two targeted epitopes are almost diametrically opposite to one another. To better understand how this translates into specific biological responses, we selected a subset of the most upregulated genes for each group (S1 for LooPINS, Fig. 5 Bi; and S2 for CaPINS, Fig. 5 B ii) and queried them against the GO Biological Process database (v. 2023) using *Enrichr* [85–87]. The five most significantly enriched GO terms (lowest P value) for each subset are presented in Fig. 5 C ii (S1) and **D.ii** (S2). ‘Loop 19-20 epitope’ actuation seems to strongly promote cell replication, with the top four terms relating to mitosis, while the fifth process relates to cell respiration, possibly indicating increased meta-bolic needs associated with the proliferative process. By contrast, four of the top GO annotations resulting from ‘Cap epitope’ actuation indicate that it chiefly favors increased expression of processes related to ECM synthesis, with collagen possibly being one of the main components (GO:0097435 “Supramolecular fiber organization”). It is also interesting to note that “Embryonic skeletal system morphogenesis” (GO:0048704) is also found in the top five terms, supporting the reported involvement of PIEZO1 mechanosensing in the differentiation processes of musculoskeletal lineages [88,89].

To further characterize the specific response for each epitope target, the top 50 differentially expressed genes (DEGs) between PIEZO1-actuated samples and the control (BINP) group are represented in the heatmaps of Fig. 5Ci (Loop 19-20) and D.i (Cap), showing the gene-level hierarchical clustering of these samples. These results clearly evidence a distinct response derived from the actuation of each epitope. In both cases, the expression pattern of the top 50 DEGs in samples cultured with the other type of PINS is closer to the control than to the stimulated group under analysis. ‘Loop 19-20’ actuation mainly affects genes related to inflammatory and proliferative processes (e.g., TGM2, CSF3), including cytokine and chemokine signaling (e.g., IL6, CXCL1/5/6). On the other hand, consistent with our previous observation, the ‘Cap’ domain favors mostly ECM-related genes, such as COL1/3/5/11/12, SERPINH1, or SPARC. Curiously, Cap actuation appears to have a stronger effect in hASC response, with the volcano plot (Fig. 5 D iii) revealing 678 significantly changed genes, of which 579 were down-regulated and 99 were upregulated. Comparatively, Loop actuation (Fig. 5C iii) generated a total of 96 DEGs, with 63 being downregulated and 33 upregulated.

Then, to attain a more global picture from these data, we carried out pre-ranked Gene Set Enrichment Analysis (GSEA) in Pathway databases. Consistent with the previous assessment, some of the signatures most positively correlated with LooPINS stimulation are ‘Regulatory circuits of the STAT3 signaling pathway’, ‘Signaling by SCF-KIT’, ‘Cytokines and inflammatory response’, or ‘Alpha-M beta-2 integrin signaling’ (Fig. 5C iv). STAT3 pathway has been chiefly associated with the regulation of cellular proliferation, apoptosis, division and differentiation [90]. It has also been associated with a role in immune response regulation and the inflammatory process [91,92]. Similarly, the stem cell factor (SCF)-KIT signaling pathway is well-known to play a crucial role in regulating cell proliferation, survival, and migration [93]. The increase of αMβ2 integrin (also known as macrophage antigen-1) signaling further reinforces the hypothesis of increased immunological and cell motility modulation [94]. Thus, the PIEZO1 Loop 19-20 activity seems to relate to an overall phenotype involving cell proliferation, migration, and inflammatory modulation. By contrast, for CaPINS samples, GSEA revealed positively correlated signatures such as ‘TGF-beta regulation of skeletal system development’, ‘Collagen biosynthesis and modifying enzymes’, or ‘Collagen chain trimerization’, corroborating earlier findings. Concomitantly, a negative correlation was found for ‘Regulation of KIT signaling’, directly contrasting with LooPINS samples (Fig. 5 D iv). Therefore, PIEZO1 Cap domain signaling appears to be responsible for a pro-anabolic, matrix-producing phenotype, possibly linked with musculoskeletal differentiation pathways.

To reinforce the evidence of these functional differences between PIEZO1 modules, we performed GSEA for a direct comparison between CaPINS and LooPINS groups. Fig. 5 E shows the Pathways upregulated by ‘Cap’ actuation and the top 10 most correlated with ‘Loop 19-20’ actuation by normalized enrichment score (NES). Once more, assembly of collagen structures is the most pronounced effect resulting from PIEZO1 signaling through the Cap domain. Interestingly, the other term associated with this group is ‘autophagy of mitochondrion’, or mitophagy. This process is responsible for the maintenance of mitochondrial quality, being essential for cell survival and homeostasis. The regulation of mitophagy is directly dependent on intracellular Ca^2+^ signaling, although the exact mechanisms by which this happens are currently under intense debate [95,96]. Our results suggest that increased intracellular Ca^2+^ from PIEZO1-based mechanotransduction via the Cap domain positively regulates mitophagy, thereby potentially promoting cellular metabolic health. Conversely, terms correlated with Loop 19-20 actuation demonstrate a strong immune response trigger, involving T cell and B cell activation or Fc-γ-dependent phagocytosis, along with the drive towards a proliferative phenotype, including transcriptional markers of increased gas transport and cell metabolism.

Finally, we narrowed down our assessment to musculoskeletal differentiation markers, looking at the impact on the differential expression of a pre-selected gene set in each epitope-actuated group. These results show an expression pattern coherent with previous findings (Fig. 5 F). Whereas the sub-panel of stemness-related markers (MKI67, GATA2 or KIT) is unanimously upregulated by LooPINS, consistent with the more proliferative/inflammatory modulation profile observed for these cells, upregulation of markers related to bone or tendon development (RUNX2, OMD, ALPL; TGFB3, GDF5) is associated with CaPINS actuation. This aligns well with the current understanding of the role of PIEZO1 in osteogenesis and bone regeneration, where it is known to promote MSC osteoblastic differentiation and bone formation through BMP, YAP, and Wnt/β-catenin signaling pathways [88,89,97,98]. This also further contributes to validating this channel as a potential therapeutic target in osteoporosis or bone fracture settings, as recently proposed with the development of a triboelectric wearable device for bone healing [99]. Similarly, a variety of fibrillar collagens and non-collagenous ECM components prevalent in tendon or bone-associated connective tissues (e.g., LUM, DCN, COMP, ASPN) are also increased in this group. Importantly, we also found an upregulation of some relevant collagen crosslinking enzymes like lysyl oxidase (LOX) or procollagen-lysine,2-oxoglutarate 5-dioxygenase 1 (PLOD1), which play key roles in tuning the mechanical properties of collagen fibrils in adaptation to external mechanical loads [100,101]. Our groups have recently reported that PIEZO1 activity increases tissue stiffness by adjusting collagen crosslinking levels, including through heightened LOX expression [20]. This finding further bolsters this association, indicating a prominent role for the Cap domain in this biological pathway.

Intriguingly, in contrast with the other musculoskeletal differentiation markers, our analysis also revealed that cartilage-related markers TGFB2, COL2A1 and ACAN were upregulated by ‘Loop 19-20’ actuation. This was rather surprising since, to date, most research on the role of PIEZOs in cartilage has supported a pro-inflammatory deleterious effect, including in the progression of osteoarthritis [102–105]. While our global results for LooPINS actuation fit relatively well in this paradigm, it was rather surprising to find concomitant pro-chondrogenic effects. A possible explanation for this apparent paradox can be found in a recent study by Nims et al. [106] The authors confirmed that PIEZO1 indeed induced a transient inflammatory profile, leading to upregulation of genes associated with cartilage degradation and osteoarthritis when activated through supraphysiologic deformation. However, under unloaded conditions, PIEZO1 was also shown to promote anabolic pro-chondrogenic effects, in another demonstration of the versatile functionality of this mechanoreceptor. Thus, the downstream impact of PIEZO1 signaling appears to depend on an array of factors, including the intensity of the applied stimulus, but also which specific subdomains are stimulated, as we demonstrate here.

## 3 Discussion

Here, we have developed a new contact-free technology for molecular-scale actuation of the mechanosensor PIEZO1 with tailored sub-molecular precision. We then proceeded to discover that actuation of specific structures of PIEZO1 elicited distinct transcriptional responses. Globally, our results indicate that the proposed molecularly imprinted nanoswitches are able to actuate targeted domains of PIEZO1 and trigger downstream signaling that is domain-specific. Our results implicate PIEZO1 signaling in a range of apparently contradictory biological processes (Fig. 5 G). In fact, the intriguing PIEZO1 multi-functionality in stem cell responses and musculoskeletal system physiology has been the focus of intense research [89,107]. It has been associated simultaneously with both catabolic and anabolic effects [89]; proliferation and maintenance of stemness properties [108], but also differentiation [97,109]; homeostasis and regeneration [110,111], as well as tissue damage and inflammatory response [112,113]. Similarly, the number and type of stimuli known to trigger PIEZO1 gating keep increasing [18,98], painting a picture of a multifunctional, versatile molecule with structure-function relationships that remain broadly to be elucidated. The large size and structural complexity of this channel also contribute to propelling this multifaceted character. Indeed, studies on the mechanisms of PIEZO1 gating have generated multiple hypotheses [25–28,37,114–117] that, instead of being contradictory, can be complementary and help to explain, to a large extent, this array of divergent downstream effects.

Two main activation models have been proposed: force-from-lipids and force-from-filament [98]. In the former, membrane surface tension creates a hydrophobic mismatch, directly causing the channel to open. In the latter, the channel is linked to specific accessory proteins and, through these, to the cytoskeleton, the action of which is responsible for channel opening. Most research so far tends to support the existence of a link between conformational shifts in the blade domains and sensing of membrane tension, arguing that PIEZO1 is inherently a force-from-lipids sensor [25,26,37,114,118]. Concomitantly, a growing body of evidence demonstrates the importance of PIEZO1 interactions with ECM components [32,33], as well as with transmembrane [29], intracellular [31], and cytoskeletal [30] proteins for regulation of its mechanosignalling behavior, also presenting the capability of responding to intracellular traction forces from the actin/myosin network [119]. These two visions are currently evolving into a combined theory where, for example, channel tethering to the ECM/cytoskeleton allows long-range, whole-cell transmission of forces sensed locally by membrane tension changes [30].

Though we cannot yet directly link the activity of the Cap domain or extracellular blade loops to any particular model, our observations fit well into this framework, suggesting that PIEZO1 mechanosensing can be triggered by stimulation of different regions in its superstructure, each generating specific cell response patterns. This differential response is a remarkable outcome of the technology developed in this study, and supports the hypothesis that PIEZO channels have multiple context-dependent activation routes and states, instead of a single “ON/OFF” functionality, such as that triggered by their pharmacological activation with Yoda1 [34]. This might help to explain their versatility, capable of sensing stimuli as different as poking, membrane tension and stretching, or fluid shear stress, deriving from both the extracellular milieu as well as the cytoskeleton [24,29]. Moreover, this finding opens a wide range of new possibilities for future PIEZO mechanosensing research that were technologically unavailable so far, where different types of mechanical stimuli can be explored toward the generation of ‘Cap-like’ or ‘Loop-like’ responses, or even a combination of both.

Another interesting finding from this study is the apparent time-dependent nature of PIEZO1 mechanosensitivity. Usually, assessment of channel activation using either mechanical (poking, stretching, or atomic force microscopy), biochemical (Yoda1), or electrical (patch clamp) stimulation is performed in very short-term experiments (up to 10 min) [25,28,37]. Interestingly, our results show that prolonged cyclical stimulation regimens are also capable of modulating PIEZO1 activity. In fact, sustained shear stress stimuli transduced by mechano-sensitive ion channels over several days have been reported to be essential for proper T cell activation and proliferation, indicating that the signaling mediated by these channels is time-dependent [120]. Also noteworthy is the apparent time-dependence of PIEZO1 effect on *YAP* gene regulation, when comparing day 1 and 7 results, along with the fact that while both LooPINS and CaPINS similarly increase YAP nuclear translocation, the downstream outcomes in gene expression are fundamentally different. These imply that discrete signaling and regulation mechanisms are simultaneously in play beyond a one-to-one sequential link between PIEZO1 activation, YAP nuclear translocation, and upregulation of target genes. This is consistent with the role of YAP as a central integrator of multiple mechanosensitive pathways, including but not limited to PIEZO1-mediated calcium signaling, such as cytoskeletal tension, actomyosin contractility, or integrin-mediated adhesion. Therefore, these results reflect a more complex and interlocked array of mechanotransduction players and inputs acting in tandem [121–123]. This could also contribute to explaining the relatively few effects of Loop actuation on the expression of assessed gene markers in ECs. This could mean that either i) the specific gene markers analyzed here are not directly altered by stimulating the Loop 19-20 region of the PIEZO1 blade, or ii) that other mechanisms parallel to YAP/TAZ signaling are concomitantly responsible for translating PIEZO1 gating into gene expression changes. In either case, this implies that different cellular components/programs are controlled by different modules of the same PIEZO1 protein. Future studies will be required to clarify these hypotheses, as well as to unequivocally show the direct link between targeted PIEZO1 magnetomechanical stimulation and YAP activity.

Due to their wireless functioning mode, the proposed nanoswitches also open novel routes for the development of targeted therapies for musculoskeletal conditions, including osteoarthritis and tendinopathy. This will logically require their combination with adequate magnetic actuation systems [40]. Considering the multifunctional nature of PIEZO1, we foresee that future developments will need to move from broad, whole-channel stimulation strategies to submolecular-directed approaches with specific mechanical stimulation regimens. In this context, magnetic manipulation of PIEZO1 using PINS offers several complementary advantages over traditional bulk contactless activation by e.g., ultrasound-based methods [124]. Ultrasound can activate mechanosensitive channels, but it does so by propagating acoustic forces that lack cell-specific targeting and may recruit multiple mechano-transduction pathways, making mechanistic interpretation more challenging. In contrast, MINP-based magnetic actuation enables molecularly targeted engagement of specific PIEZO1 domains, allowing force delivery at defined subcellular locations with high spatial precision. Additionally, magnetic fields can be applied with fine temporal control and minimal off-target mechanical perturbation, and they are inherently non-invasive and compatible with simultaneous high-resolution optical readouts. These features make magnetic nano-switches a powerful tool for dissecting the mechanobiology of PIEZO1 and for achieving cell-type selective mechanogenetic modulation that is difficult to achieve with bulk methods alone.

Possible stem cell-based therapies will also need to consider how different microenvironmental cues synergistically contribute to determining cell fate decisions. Cell behavior, including fate commitment, is well known to be influenced by the integration of multiple cues from their surroundings [125–127]. This includes biophysical cues, such as ECM mechanical properties, three-dimensional organization, or external forces, where the PIEZO1 mechanosensing response has been acquiring an increasingly prominent role [128]; but also soluble biological cues, like growth factors, hormones, or small chemicals. Thus, the use of representative *in vitro* models, incorporating 3D biomimetic scaffolds and biochemical stimuli in tandem, will allow the study of PIEZO1 roles in different pathophysiological processes to better inform the development of more effective therapies [129,130]. Also envisioning potential therapies, future studies will be required to systematically assess and optimize nanoparticle formulations with respect to long-term stability, biodistribution, and biosafety to support their eventual translation toward *in vivo* applications, particularly considering the iron oxide contents of the nanoparticles.

Ultimately, the expansion of the proposed concept to other PIEZO protein domains and their time-magnitude dependent actuation might lead to further breakthroughs in deciphering the structure-function relationships of this important mechanoreceptor. This is particularly attractive for the exploration of protein regions with molecular structures still unresolved, such as the distal blades [26,28]. These regions can have unknown functional roles, for example, on the phenomenon of crowding-induced opening of PIEZO1 [115], or on its participation in cell-cell junctions through interaction with Platelet Endothelial Cell Adhesion Molecule 1 (PECAM1) and Cadherin-5 [29]. In this regard, the proposed molecular imprinting-based concept holds tremendous promise, since it allows a rapid, straightforward design adaptation to produce new nanoactuators targeting specific domains of interest, with short development times and low experimental investment [46–49]. Thus, we believe the proposed wireless nanoswitches have the potential to transform the way in which we study PIEZO-mediated mechanobiology, providing a new tool with submolecular precision to unveil the fundamental mechanics and mechanisms of activity of these intricate proteins, generating functional knowledge that can be leveraged to unlock their therapeutic potential.

Although this study reveals clear domain-dependent effects of PIEZO1 actuation across multiple cellular contexts, the analysis of human adipose tissue–derived stem cells is primarily based on transcriptomic profiling and does not include direct protein-level or functional validation of differentiation outcomes. We have prioritized an unbiased transcriptomic strategy, allowing comprehensive identification of global, domain-specific regulatory programs elicited by selective PIEZO1 actuation – information that would not be accessible through targeted assays alone. However, future studies will be required to functionally validate specific lineage commitment outcomes and, subs-equently, confirm the translational potential of PINS for stem cell differentiation manipulation. Nonetheless, the present data already establish a robust molecular framework linking subdomain-specific PIEZO1 engagement to distinct transcriptional responses in hASCs, providing a strong basis for further developments.

Overall, our work also fits within a rapidly advancing field in which molecular imprinting technology is being repurposed from analytical and sensing applications toward precisely engineered biomedical interfaces [131]. In this broader context, molecularly imprinted polymers are increasingly recognized not only for their high selectivity and stability but also for their ability to act as synthetic recognition elements that mimic natural receptors in complex biological environments. Recent reviews highlight the expanding utility of MIPs, from diagnostic biosensors and drug delivery systems to engineered biomaterials with tailored bioactivity, pointing to emerging opportunities for imprinting strategies that target biologically relevant structures such as proteins and cellular receptors. Embedding molecular specificity directly into functional nanomaterials, as we do with PINS, exemplifies how molecular imprinting is evolving into a versatile platform for precision mechanobiology and targeted biomedical modulation beyond conventional sensing paradigms.

## 4 Conclusions

In this work, we have developed MINPs with affinity for specific functionally relevant regions of the PIEZO1 mechanoreceptor, whose signaling mechanisms remain a significant research challenge, by integration of *in silico* analysis and the available knowledge on its structure-function relationship. These PINS were designed to incorporate different functionalities, namely superparamagnetic response and fluorescence. Fluorescent PINS were shown to function as “synthetic antibodies” enabling PIEZO1 immunolabeling *in vitro*. More notably, we demonstrated that remote magnetomechanical actuation of PIEZO1 through its interaction with magnetically-responsive PINS led to changes in the expression patterns of genes downstream of PIEZO1 activity, thereby demonstrating the functionality of magnetic MINPs as wireless, tailor-made “nanoswitches”. Furthermore, the resulting gene expression patterns were shown to be dependent on the targeted PIEZO1 domain, implying separate roles for these substructures in the intricate signaling functionality of the receptors. While the actuation of the extracellular Cap domain was associated with a pro-anabolic, musculoskeletal differentiation and matrix deposition phenotype, stimulation of the Loop 19-20 epitope leads to a more proliferative, stem-like response, with prevalent modulation of inflammatory markers. These differential downstream responses support the hypothesis of PIEZO1 as a multifaceted protein with more than one activation route, helping to explain its capability to translate a wide array of mechanical stimuli into different signaling cascades.

Altogether, the proposed technology holds promising potential as a tool for studying PIEZO-mediated mechanobiology, allowing the highly selective targeting of specific regions of interest in its superstructure. Moreover, considering the ability of PINS to wirelessly modulate gene expression in stem cells, we envision its potential application as innovative nanomedicines for promoting regenerative responses in different mechanosensitive tissues. In particular, musculoskeletal tissues known to be responsive to these stimuli, such as tendons, ligaments, cartilage or bone, could benefit from these novel nanoswitches.

## 5 Experimental section

### Reagents and Materials

Unless otherwise noted, all reagents and materials were acquired from Sigma-Aldrich (Merck KGaA, Germany)

### PIEZO1 epitope selection

A whole-protein bioinformatic workflow called *fingerprint analysis* was followed to predict suitable epitopes for imprinting [46,52]. To that end, the target protein sequence in humans was extracted from the UniProt database [53] - Q92508 (PIEZ1_HU-MAN). This sequence was inserted into ExPASy PeptideCutter [132,133] and cut using only Trypsin. Resulting peptides with fewer than 8 amino acids were discarded since they tend to have low specificity, while those over 15 amino acids can lead to undesired problems in the imprinting process [46]. Remaining peptides between 8 and 15 amino acids (including) were aligned against the UniProtKB and SwissProt databases using BLASTP [134] and classified by S and E values (Table S1, Supporting Information). Based on the available annotations in the UniProt sequence, peptides that corresponded to transmembrane helical regions or to the intracellular beam and associated regions were also excluded due to their inaccessibility. The remaining sequences with the highest S and lowest E scores were then visualized using AlphaFold Protein Structure Database (EMBL-EBI, Cambridge, UK) to determine their location in the protein 3D structure [54,55]. The peptides found to be located in relevant extracellular domains of PIEZO1 were finally selected for synthesis.

### Zinc-doped superparamagnetic iron oxide nanoparticle (Zn-SPION) synthesis

Syntheses were carried out in a 100-mL three-neck round-bottom flask equipped with a water-cooled condenser connected to a standard Schlenk line, following previously reported protocols for SPION preparation with some modifications [57]. In brief, spherical-shaped Zn-SPIONs were synthesized by mixing iron(III) acetylacetonate (2.4 mmol, 0.84 g), zinc (II) acetylacetonate (0.6 mmol, 0.16 g) and 1,2-hexadecanediol (10 mmol, 2.87 g) in the presence of oleic acid (OA; 6 mmol, 1.88 g), oleylamine (OAm; 6 mmol, 1.89 g), and benzyl ether (20 mL) under magnetic stirring. The mixture was degassed for 1 h under vacuum at 110 °C. Under nitrogen flow, the temperature was increased up to 210 °C at a heating rate of 8 °C min^−1^ and the solution was allowed to react at 210 °C for 2 h. Then, the temperature was increased at 5 °C min^−1^ until 295 °C, and the reaction was kept at reflux for 1 h. The reaction was stopped by removing the heating mantle, and the mixture was cooled down to room temperature (RT) for 2 h. After the addition of 50 mL of ethanol, a black precipitate was obtained by centrifugation (4000 rpm, 10 min). The supernatant was then removed, and the precipitate was resuspended in 20 mL of toluene mixed with 0.03 mL of OA and 0.03 mL of OAm. The solution was centrifuged (3000 rpm, 10 min) and the precipitate discarded to remove aggregates. Finally, 50 mL of ethanol was added to the supernatant, and the solution was centrifuged again (4000 rpm, 10 min). The collected Zn-SPIONs were dispersed in chloroform and stored at 4 °C.

### Zn-SPION coating with hydrophilic polymer

Since the synthesized Zn-SPIONs were stable only in organic media, an additional step was required for their transfer to aqueous media. This was carried out by means of a polymer coating technique using a dodecylamine-modified poly-(isobutylene-*alt*-maleic anhydride) polymer (PMA). This polymeric complex was synthesized following a previously reported protocol [57]. The coating process was carried out by mixing the dodecylamine-PMA complex and Zn-SPIONs, both resuspended in chloroform, in a round-bottom flask at a ratio of X = 400 nm^−2^, where X corresponds to the number of PMA monomers per effective Zn-SPION surface unit area. After mixing, the solvent was slowly evaporated with a rotavapor at 200 rpm, 45 °C, and 450 mbar until the sample was dry. The solid film formed in the flask was redissolved in 30 mL of chloroform, and the evaporation process was repeated twice more. Then, the final product was dissolved in sodium borate buffer, 50 mM, pH 12, under vigorous stirring for 1 h, with the sample being concentrated by centrifugal filtration (MWCO 30 kDa) at 2000 rpm and subsequently transferred into ultrapure (UP) water.

### Solid phase molecular imprinting of nanoparticles (MINPs)

The Protocol followed for the synthesis of MINPs was adapted from Refs. [60,135] and is described in further detail in the Supporting Information. First, glass bead surfaces were silanized with either (3-aminopropyl) trimethoxysilane or (3-glycidyloxypropyl) trimethoxysilane 2 % (v/v) in anhydrous toluene (Carlo Erba, France) for 24 h at room temperature (RT), for coupling with biotin or PIEZO1 epitopes, respectively. For biotin immobilization, amine-functionalized beads were incubated overnight in PBS pH 7.4 (0.4 mL/g beads) containing 0.5 g L^−1^ biotin, 10 g L^−1^ N-(3-dimethylaminopropyl)-N′-ethylcarbodiimide hydrochloride (EDC), and 15 g L^−1^ N-hydroxysuccinimide (NHS). For PIEZO1 epitope immobilization, epoxy-functionalized beads were incubated overnight in 10 mM carbonate buffer pH 9.0 (1 mL/g beads) containing 0.2 g L^−1^ epitope (custom production by GeneCust, France), followed by washing several times in the same buffers. Both peptides contained a terminal lysine to ensure their oriented immobilization on the glass bead surfaces. Unreacted groups were blocked with 1 M ethanolamine, pH 9.0 (3 mL/g beads) for 2 h, followed by washing in the same buffer. Finally, beads were rinsed with deionized water and dried under vacuum. Confirmation of bead functionalization was performed by fluorescein labeling as previously described [135].

For MINP polymerization, *N*-isopropylacrylamide (3.4 mM), N,N′-methylenebisacrylamide (0.13 mM), *N*-*tert*-butylacrylamide (2.6 mM), acrylic acid (0.32 mM) and *N*-(3-aminopropyl) methacrylamide hydrochloride (0.33 mM) were dissolved in ultrapure (UP) water. 2 mg of hydrophilic Zn-SPIONs were added to the solution and thoroughly mixed. To produce fluorescent MINPs for microscopy visualization, fluorescein *O*-methacrylate (0.13 mM) was added to the previous solution. The solution was degassed by sonicating under vacuum using a Sonics VCX-130PB-220 (Sonics & Materials, USA) for 15 min, followed by purging with N_2_ for 30 min. Template-derivatized glass beads were degassed by performing three cycles of alternate vacuum/N_2_. The monomer solution was poured onto the beads while gently hand swirling, and flushing of the flask with N_2_ was continued for 5-10 min. Polymerization was triggered by adding ammonium persulfate (0.26 M) and tetramethylethylenediamine (0.66 M) into the bulk of the mixture, and it was then left to react for 2 h at RT under N_2_ flow, with periodic swirling.

After synthesis, the mixture was transferred to a solid phase extraction tube fitted with a 20-μm porosity polyethylene frit. The solution was removed by vacuum filtration and then replaced with 30 mL of fresh UP water at RT. Unreacted monomers and low-affinity polymers were removed by repeating this process nine times. Then, 30 mL of UP water (at 70 °C) was added to the beads, and the tube was placed in a water bath at 70 °C for 15 min. After incubation, MINPs were collected by applying a vacuum. This incubation step was repeated 3 times, for 2 min each. The collected MINP suspension was concentrated by ultrafiltration using Vivaspin 20 Ultrafiltration Unit (30 kDa molecular weight cutoff; Sartorius AG, Germany), performing consecutive centrifugations at 15 °C for 15 min at 3200×*g*, until it was reduced to the final desired volume. The resulting suspension was freeze-dried, and the yield was calculated by weighing the dry nanoparticles.

### Size and surface charge distribution of nanoparticles

Nanoparticle (MINPs and Zn-SPIONs) size and surface charge distribution were analyzed by dynamic/electrophoretic light scattering (DLS/ELS) after ultrafiltration and before lyophilizing, using a Zetasizer Nano ZS equipment (Malvern Panalytical, Spectris plc, UK) equipped with a He-Ne laser operating at 4.0 mW and 633 nm. 1 mL aliquot of suspension was transferred into a disposable polystyrene cuvette and loaded into the equipment. Measurement parameters were automatically determined by the equipment, and 3 measurements were recorded per sample. To measure zeta potential, a Malvern Universal ‘Dip’ Cell was inserted into the polystyrene cuvette according to manufacturer instructions.

### Transmission electron microscopy visualization of NPs

A Phillips CM-12 microscope operating at 120 kV was used to visualize NPs. Briefly, a droplet of NP suspension was placed on a TEM Grid - Ultrathin Carbon Film on Lacey Carbon Support Film, 400 mesh, Copper (TED PELLA, USA), and dried at room temperature for 1 h.

### Magnetic behavior of NPs

NP magnetic characterization was carried out with a Superconducting Quantum Interference Device (SQUID; Quantum Designs MPMS5, San Diego, CA). Magnetization curves were measured from - 4 to 4 T at 300 K. The analyzed samples were dried under vacuum for 48 h, then using 5-10 mg for each measurement, normalizing the obtained magnetization values to this weight.

### Determination of MINP affinity for template epitope by SPR

Biacore X100 (Cytiva, Sweden) was used throughout the following procedures, which are adapted from Ref. [135] and described in further detail in the Supporting Information. Sensor chip CM5 (Cytiva, Sweden) was used to immobilize PIEZO1 epitopes. First, the best pH was scouted to maximize ligand immobilization. PIEZO1 Cap epitope was diluted to 2.5 mg mL^−1^ in 10 mM acetate buffer at pH 3.5, 4.0, 4.5, 5.0, and 5.5, with maximal response achieved at pH 4.5. PIEZO1 Loop 19-20 epitope was diluted to 5 mg mL^−1^ in 10 mM acetate buffer at pH 5, 5.5, 6, 6.5, 7, 8, and 9, with maximal response attained at pH 6. These specifications were then used for immobilization of epitopes onto the experimental flow cell (Fc2) using carbodiimide chemistry, while blank immobilization was performed on the reference flow cell (Fc1), by loading it with a mixture of EDC (75 g L^−1^; Cytiva) and NHS (11.5 g L^−1^; Cytiva), followed by ethanolamine HCl pH 8.5 (1.0 M, Cytiva). Finally, a regeneration buffer was injected five times to remove loosely bound molecules.

To assess MINP interaction with the template epitope, the Biacore single-cycle kinetics standard procedure was followed, consisting of five successive injections at increasing concentrations onto the sensor chip. Two blank cycles were performed using the same settings but injecting running buffer instead of MINP samples. MINPs were injected at increasing concentrations for 120 s each, with 600 s of dissociation time after the last injection. A single injection of regeneration buffer for 120 s was performed to clean the sensor surface at the end of the run. The resulting sensorgram was analyzed using Biacore X100 Evaluation Software™. Reference curves (Fc1) were automatically subtracted from each active sensorgram. The average of the two blank cycles was then also subtracted from the test run sensorgram. The subtracted curves were finally fitted using a 1:1 binding kinetics model. Three replicates were performed for each MINP type.

### Cell cultures

Two cell types were used throughout this work. Human vascular endothelial cells – ECs (EA.hy926 cell line, CRL-2922, ATCC, USA) were plated in complete Dulbecco’s Modified Eagle Minimum Essential Medium (Thermo Fisher Scientific, USA) (supplemented with 10 % fetal bovine serum and 1 % antibiotic/antimycotic) and incubated in a humidified atmosphere at 37 °C with 5 % CO_2_. Human adipose-derived stem cells (hASCs) were isolated from lipoaspirates according to a standard protocol with collagenase digestion [136]. Human liposuction aspirates were obtained from healthy female anonymous donors under informed consent and according to protocols approved by the Ethical Committee of Hospital da Prelada (Porto, Portugal, Approval No. 005/2019). Cells were plated in complete Alpha-Modified Eagle Minimum Essential Medium (Thermo Fisher Scientific, USA) and incubated in a humidified atmosphere at 37 °C with 5 % CO_2_. Culture medium was replaced every 3 days, and cells were subcultured at 80 % confluence. Cells up to passage number 5 were used for *in vitro* experiments.

### In vitro *assessment of MINP binding to cells*

ECs were seeded on 13-mm coverslips in a 24-well non-adherent plate at a density of 5 × 10^3^ cells per coverslip. After allowing cells to grow for three days, the media was removed, and cells were fixed in 10 % neutral buffered formalin (Thermo Fisher Scientific, USA). Fixed cells were rinsed with PBS, blocked for 1 h at room temperature (RT) with 5 % (w/v) bovine serum albumin in PBS, and incubated with either green fluorescent MINPs (0.0228 g L^−1^) or anti-PIEZO1 antibody (1 μg mL^−1^, 15939-1-AP, Thermo Fisher, USA, lot no. 00121356) diluted in blocking buffer overnight at 4 °C on an orbital shaker. ECs incubated with primary antibody were rinsed in PBS and incubated 1 h at RT with the secondary antibody (A21206, Thermo Fisher, USA, lot no. 2668665) diluted in PBS (2 μg mL^−1^). ECs were then rinsed thoroughly and counterstained with 4,6-diamidino-2-phenyindole (DAPI, 1 μg mL^−1^ in PBS) before observation in a Zeiss LSM 980 confocal laser scanning microscope.

### Widefield total internal reflection fluorescence (TIRF) microscopy

Experiments were performed on a Nikon Ti-E widefield TIRF microscope equipped with a 488 nm laser (Agilent Monolithic Laser Combiner MLC400B), with the power set between 300 and 450 μW at the sample to minimize photobleaching. The TIRF excitation angle was adjusted by changing the excitation angle into the TIRF oil immersion lens (Nikon Plan Apo TIRF 60x Oil DIC H N_2_) at 60x magnification and 1.4 NA, using the motorized mirror of the TIRF system. A suited filter cube was used to block the 488 nm laser line (LF488 - Ex 482/18 nm EM 525/25 nm). Signal was recorded with an Andor IXon Ultra 897 EM-CCD camera (Andor DU-897 X-9366), with activated EM gain set to 250 and with EM Chip read out of 10 MHz at 16bit (512px X 512px).

For imaging, cells were seeded in 35 mm glass bottom dishes (Ibidi). Experiments were performed at room temperature without environmental control, with cells kept in [Ca^2+^]_i_-assay buffer, composed of Hank’s Balanced Salt Solution (HBSS), with calcium, magnesium, no phenol red (Gibco) supplemented with 0.02 M HEPES (N-2-hydroxyethylpiperazine-N-2-ethane sulfonic acid) (Gibco). Fluo-4 staining was performed according to Refs. [20,67] to allow visualization of calcium influx. Prior to imaging, culture media was removed and cells rinsed gently (3x) with [Ca^2+^]_i_-assay buffer. Fluo-4 (Invitrogen, F14217) was diluted to a final concentration of 5 μM in [Ca^2+^]_i_-assay buffer after mixing with Pluronic F-127 (Thermo Fisher, P3000MP; final concentration 0.02 % w/v) and then added to the cells and incubated for 25 min at 37 °C followed by 35 min at RT. It was then removed and the cells rinsed (3x) with [Ca^2+^]_i_-assay buffer. Cells were kept in the same buffer at 37 °C until TIRF imaging, allowing for >30 min for de-esterification. As a negative control, ECs were incubated with 5 μM GsMTx4 (Merck, SML3140) in assay buffer for 30 min at 37 °C. Time lapse recordings were acquired within 1 h following GsMTx4 incubation to ensure PIEZO channel inhibition.

Widefield TIRF measurements were taken on fields of view (FOVs) of about 140 by 140 mm (Calibration 0.27mm/px), with an integration time of 50 ms per frame. Time-lapse recordings were taken at 100 ms intervals, over time windows of 1 min duration. For magnetic particle activation, a tower of small resizing permanent magnets made of neodymium iron boron (Supermagnete, Germany; Hyab Magneter AB, Sweden) was built according to Ref. [137]. The maximum magnetic field as measured at its tip with a Gaussmeter was approx. 0.110 T. About 20 s after starting the recording, the tip was approached to the sample until the glass bottom was reached close to the laser illumination area without touching the cells. The time stamp could be used to analyze the intensity evolution during an observation of 10 s after stimulation. For every condition (No MINPs, LooPINs, and CaPINS) FOVs with multiple cells were analyzed and recorded with or without magnet activation by the magnetic probe.

Fiji was used for image processing. To analyze the intensity variations over time, regions of interest of each cell located completely within the FOV were extracted using a 125 x 125 px square. Intensity variations were analyzed in dependence of magnetic actuation and type of PINS applied. The activation time point was identified in the video and the subsequent 10 s acquisition time window measured in terms of intensity evolution. The variation in fluorescence intensity at each timepoint (ΔF) in proportion to the baseline set at the moment of magnetic stimulation (F_0_), i.e., ΔF/F_0_ ratio, was finally plotted over the acquisition time (s).

### Continuous magnetic actuation on Piezo1 mechanoreceptors

Either ECs or hASCs were seeded on 13-mm coverslips in a 24-well non-adherent plate at a density of 5 × 10^3^ cells per coverslip. After allowing cells to adhere overnight, they were incubated with magnetic MINPs – either BINPs, LooPINS, or CaPINS (0.0228 g L^−1^ in the respective culture medium) – for 6 h on an orbital shaker. Media containing MINPs was then removed, cells were rinsed twice with fresh media to remove MINPs that were adsorbed loosely or unspecifically, and new media was added. Day 0 samples were immediately collected by immersing coverslips in TRIzol reagent (Thermo Fisher Scientific, USA; 0.8 mL per coverslip) and frozen at − 80 °C to serve as normalization controls in quantitative real-time polymerase chain reaction (qPCR) experiments. The remaining cells were cultured for either 1 or 7 days in a humidified atmosphere at 37 °C with 5 % CO_2_, either with or without magnetic stimulation. To apply the magnetic stimulus, a Magnefect-LT device (nanoTherics Ltd., UK) fitted with 24-magnet arrays was used as recommended by the manufacturer (Figure S4 A, Supporting Information). A displacement of 0.2 mm at 2 Hz was applied throughout the culture period. At the specified time point, cells were collected in TRIzol reagent and frozen at − 80 °C until further processing.

### Piezo1 knockdown

Accell Piezo1 siRNA was purchased from Dharmacon (A-020870-13-0020) and reconstituted to 100 μM in 5x siRNA Buffer (Dharmacon, B-002000-UB-100). Likewise, Accell Non-Targeting siRNA (Dharmacon, D-001910-10-20) was reconstituted in the same buffer to the same concentration. To make inoculation media, serum and antibiotic free High Glucose Dulbecco’s Modified Eagle Medium (Gibco, 11965092) was mixed with either siRNA to a final concentration of 1 μM. EA.hy926 cells were plated at 3000 cells/cm2 in 24 well plates and left to attach overnight in complete culture media. The media was removed and the cells were washed twice with PBS to remove any remaining serum. 200 μL of Piezo1 siRNA was put in 12 wells and 200 μL of Non-Targeting siRNA in the other 12 wells. The plates were then placed in a 37 °C, 5 % CO_2_ incubator for 144 h 50 μL of DMEM without serum or antibiotics was added to each well on day 3 and 5 to provide glucose and maintain media coverage. The remaining procedures for MINP incubation and magnetic stimulation were the same as the ones performed for wild-type cells.

### Magnetic field simulations

Finite Element Method Magnetics (FEMM) Version 4.2 (28Feb2018 Build) was applied to simulate the magnetic field produced by the Magnefect-LT device used in this study [138]. Problem Type was set to ‘Planar’, and the remaining parameters were accepted as automatically filled in by the software. A detailed description of the problem drawing is provided in the Supporting Information (Materials and Methods and Figure S4 B). The mesh was generated using standard parameters, and results were then analyzed, as shown in Figure S4 C&D.

### Calculation of magnetic force applied on nanoswitches

The detailed assumptions and calculations for estimating the magnetic force applied on PIEZO1 nanoswitches by the wireless actuation setup are provided in the Supporting Information.

### RNA extraction and qPCR assessment of gene expression

Samples frozen in TRIzol were thawed to RT, and total RNA was extracted following the optimized protocol developed by Toni et al. (2018) [139]. RNA concentration and purity were assessed using a NanoDrop ND-1000 spectrophotometer (Thermo Fisher Scientific, USA). Total RNA was reverse transcribed using qScript™ cDNA Synthesis Kit (Quanta Biosciences, USA). qPCR was performed using PerfeCTA® SYBR Green FastMix (Quanta Biosciences, USA), according to the manufacturer’s protocol, and run on a QuantStudio5 thermocycler (Applied Biosystems, Thermo Fisher Scientific, USA). Primer sequences (Table S9, Supporting Information) were designed using the Primer-BLAST NCBI tool and synthesized by Eurofins Genomics (Luxembourg).

Melting curves were analyzed at the end of every reaction to verify the specificity of PCR products. 2^−ΔΔCt^ method was applied to calculate relative gene expression [140]. Glyceraldehyde-3-phosphate dehydrogenase gene (*GAPDH*) was chosen as the reference gene, owing to the stability of its expression across the sample sets. All values were first normalized to average transcript levels of *GAPDH*, and then to gene expression in cells collected on day 0, as given by Equation (1):

(1) $$R=2^{-[\text{Δ}Ct\;sample\;-\text{Δ}Ct\;control\;]}$$

*R* being the relative expression ratio of each gene of interest (GOI), Δ*Ct sample* is the difference between the crossing points (Ct) of GOI and *GAPDH* (reference gene) in each sample, and Δ*Ct control* is the difference between the Ct of GOI and *GAPDH* in the control group (day 0 samples).

### Genome-Wide RNA Sequencing (RNA-Seq)

RNA-seq was performed on Day 7 hASC samples. RNA-Seq libraries construction and sequencing were carried out by Azenta/GENEWIZ (Leipzig, Germany). Briefly, mRNA integrity and yield were assessed with NanoDrop (ThermoFisher, USA), with samples required to have A260/A280 = 1.8-2.2. RNA-Seq libraries were prepared with poly-A selection enrichment and sequenced using Illumina NovaSeq technology with 2 × 150–base pair (bp) read length to a sequencing depth of at least 20 million reads.

### Data Preprocessing and Bioinformatics Analysis

Primary-level bioinformatics analysis was carried out using the R package ezRun, which is implemented within the SUSHI framework of the Functional Genomics Center Zurich (ETH Zurich and the University of Zurich) [141]. Quality of the raw RNA-seq data was verified with FastQC and FastQ Screen (v0.15.3) [142]. Raw.fastq files were mapped to the human genome reference (Ensembl GRCh38.p10 – Release_42-2023-01-30) with the STAR aligner software [143]. Differential expression analysis was performed by using the kallisto software using default parameters [144]. Principal component analysis (PCA) and heatmap renderings were performed with the ClustVis tool [145]. For analysis of gene expression signatures, raw read counts were imported into Omics Playground Suite (BigOmics Analytics, Switzerland) and implemented in RStudio (v. 1.3.1093) [146]. Volcano plots were visualized using the VolcaNoseR package [147]. Pathway enrichment analysis of differentially expressed genes or selected subclusters of genes of interest was carried out using the corresponding Enrichr tool [85]. Gene lists were interrogated against the Gene Ontology Biological Process 2023 database. Gene set functional enrichment was performed by pre-ranked Gene Set Enrichment Analysis (GSEA) using fgsea [v1.16.0] [148], with the corresponding plots rendered using Omics Playground. Analyses were performed on both upregulated and down-regulated genes, unless specified otherwise in the figure legends.

### Statistical analysis

GraphPad Prism 9.5.1 was used to perform statistical analysis and draw the graphs displayed in Fig. 2C and D, 3 B & C, 4 A, B & C, and 5 C.ii, D.ii, E & F. Outliers were identified and removed using the Robust regression and Outlier removal (ROUT) method, with Q = 1 %. Either one-way (MINP size and surface charge distribution, MINP fluorescence level on ECs, YAP nuclear translocation) or two-way (gene expression) analysis of variance (ANOVA) were performed after confirming the sample normality with D’Agostino-Pearson omnibus test, followed by Tukey’s post-hoc t-tests for individual comparisons. When samples did not pass the normality test (calcium fluorescence intensity), Kruskal-Wallis test, followed by Dunn’s test for individual comparisons was used instead. Significance level was set to α < 0.05; unless otherwise noted, *p < 0.05, **p < 0.01, ***p < 0.001, ****p < 0.0001.

## Supplementary Material

Supplementary data to this article can be found online at https://doi.org/10.1016/j.bioactmat.2026.03.051

Supplementary data

Video 1

Video 2

Video 3

## Figures and Tables

**Table 1:** Values calculated for kinetic constants derived from single cycle kinetic (SCK) assays (n = 3) and corresponding equilibrium dissociation constant (*K*_*D*_ = *k*_*d*_*/k*_*a*_). Data presented as mean ± SD.

Sample	*k_a_* (M^–1^ s^–1^)	*k_d_* (s^–1^)	*K_D_* (M)
LooPINS	(1.5 ± 1.0) × 10^6^	(4.5 ± 2.0) × 10^–4^	(3.0 ± 2.4) × 10^–10^
CaPINS	(1.7 ± 2.0) × 10^6^	(1.3 ± 0.3) × 10^–4^	(7.6 ± 9.3) × 10^–10^

## Data Availability

The main data supporting the findings of this study are available within the paper and its Supplementary Information. The raw and analyzed datasets generated during the study are available from the corresponding author on reasonable request.

## References

[1] Du H, Bartleson JM, Butenko S, Alonso V, Liu WF, Winer DA, Butte MJ (2023). Tuning immunity through tissue mechanotransduction. Nat Rev Immunol.

[2] Lavagnino M, Wall ME, Little D, Banes AJ, Guilak F, Arnoczky SP (2015). Tendon mechanobiology: Current knowledge and future research opportunities. J Orthop Res.

[3] Ferrai C, Schulte C (2024). Mechanotransduction in stem cells, Eur. J Cell Biol.

[4] Guimarães CF, Gasperini L, Marques AP, Reis RL (2020). The stiffness of living tissues and its implications for tissue engineering. Nat Rev Mater.

[5] Inguito KL, Schofield MM, Faghri AD, Bloom ET, Heino M, West VC, Ebron KMM, Elliott DM, Parreno J (2022). Stress deprivation of tendon explants or Tpm3.1 inhibition in tendon cells reduces F-actin to promote a tendinosis-like phenotype. Mol Biol Cell.

[6] Heo S-J, Thakur S, Chen X, Loebel C, Xia B, McBeath R, Burdick JA, Shenoy VB, Mauck RL, Lakadamyali M (2022). Aberrant chromatin reorganization in cells from diseased fibrous connective tissue in response to altered chemomechanical cues. Nat Biomed Eng.

[7] Tassinari R, Olivi E, Cavallini C, Taglioli V, Zannini C, Marcuzzi M, Fedchenko O, Ventura C (2023). Mechanobiology: A landscape for reinterpreting stem cell heterogeneity and regenerative potential in diseased tissues. iScience.

[8] Saraswathibhatla A, Indana D, Chaudhuri O (2023). Cell–extracellular matrix mechanotransduction in 3D. Nat Rev Mol Cell Biol.

[9] Di X, Gao X, Peng L, Ai J, Jin X, Qi S, Li H, Wang K, Luo D (2023). Cellular mechanotransduction in health and diseases: from molecular mechanism to therapeutic targets, Signal Transduct. Targeted Ther.

[10] Xiao B (2020). Levering mechanically activated Piezo channels for potential pharmacological intervention. Annu Rev Pharmacol Toxicol.

[11] Woo S-H, Lukacs V, De Nooij JC, Zaytseva D, Criddle CR, Francisco A, Jessell TM, Wilkinson KA, Patapoutian A (2015). Piezo2 is the principal mechanotransduction channel for proprioception. Nat Neurosci.

[12] Ranade SS, Woo S-H, Dubin AE, Moshourab RA, Wetzel C, Petrus M, Mathur J, Bégay V, Coste B, Mainquist J, Wilson AJ (2014). Piezo2 is the major transducer of mechanical forces for touch sensation in mice. Nature.

[13] Douguet D, Patel A, Xu A, Vanhoutte PM, Honoré E (2019). Piezo ion channels in cardiovascular mechanobiology. Trends Pharmacol Sci.

[14] Beech DJ, Kalli AC (2019). Force sensing by Piezo channels in cardiovascular health and disease. Arterioscler Thromb Vasc Biol.

[15] Bornstein B, Konstantin N, Alessandro C, Tresch MC, Zelzer E (2021). More than movement: the proprioceptive system as a new regulator of musculoskeletal biology. Curr Opin Physiol.

[16] Dance A (2020). The quest to decipher how the body’s cells sense touch. Nature.

[17] Qin L, He T, Chen S, Yang D, Yi W, Cao H, Xiao G (2021). Roles of mechanosensitive channel Piezo1/2 proteins in skeleton and other tissues. Bone Res.

[18] Wu J, Lewis AH, Grandl J (2017). Touch, tension, and transduction – The function and regulation of Piezo ion channels. Trends Biochem Sci.

[19] Götschi T, Held V, Klucker G, Niederöst B, Aagaard P, Spörri J, Passini FS, Snedeker JG (2023). PIEZO1 gain-of-function gene variant is associated with elevated tendon stiffness in humans. J Appl Physiol.

[20] Passini FS, Jaeger PK, Saab AS, Hanlon S, Chittim NA, Arlt MJ, Ferrari KD, Haenni D, Caprara S, Bollhalder M, Niederöst B (2021). Shear-stress sensing by PIEZO1 regulates tendon stiffness in rodents and influences jumping performance in humans. Nat Biomed Eng.

[21] Nakamichi R, Ma S, Nonoyama T, Chiba T, Kurimoto R, Ohzono H, Olmer M, Shukunami C, Fuku N, Wang G, Morrison E (2022). The mechanosensitive ion channel PIEZO1 is expressed in tendons and regulates physical performance. Sci Transl Med.

[22] Wang L, You X, Lotinun S, Zhang L, Wu N, Zou W (2020). Mechanical sensing protein PIEZO1 regulates bone homeostasis via osteoblast-osteoclast crosstalk. Nat Commun.

[23] Gan D, Tao C, Jin X, Wu X, Yan Q, Zhong Y, Jia Q, Wu L, Huo S, Qin L, Xiao G (2024). Piezo1 activation accelerates osteoarthritis progression and the targeted therapy effect of artemisinin. J Adv Res.

[24] Saotome K, Murthy SE, Kefauver JM, Whitwam T, Patapoutian A, Ward AB (2018). Structure of the mechanically activated ion channel Piezo1. Nature.

[25] Zhao Q, Zhou H, Chi S, Wang Y, Wang J, Geng J, Wu K, Liu W, Zhang T, Dong MQ, Wang J (2018). Structure and mechanogating mechanism of the Piezo1 channel. Nature.

[26] Yang X, Lin C, Chen X, Li S, Li X, Xiao B (2022). Structure deformation and curvature sensing of PIEZO1 in lipid membranes. Nature.

[27] Lewis AH, Grandl J (2020). Inactivation kinetics and mechanical gating of Piezo1 ion channels depend on subdomains within the cap. Cell Rep.

[28] Mulhall EM, Gharpure A, Lee RM, Dubin AE, Aaron JS, Marshall KL, Spencer KR, Reiche MA, Henderson SC, Chew T-L, Patapoutian A (2023). Direct observation of the conformational states of PIEZO1. Nature.

[29] Chuntharpursat-Bon E, Povstyan OV, Ludlow MJ, Carrier DJ, Debant M, Shi J, Gaunt HJ, Bauer CC, Curd A, Simon Futers T, Baxter PD (2023). PIEZO1 and PECAM1 interact at cell-cell junctions and partner in endothelial force sensing. Commun Biol.

[30] Wang J, Jiang J, Yang X, Zhou G, Wang L, Xiao B (2022). Tethering Piezo channels to the actin cytoskeleton for mechanogating via the cadherin-β-catenin mechanotransduction complex. Cell Rep.

[31] Zhou Z, Ma X, Lin Y, Cheng D, Bavi N, Secker GA, Li JV, Janbandhu V, Sutton DL, Scott HS, Yao M (2023). MyoD-family inhibitor proteins act as auxiliary subunits of Piezo channels. Science.

[32] Lai A, Thurgood P, Cox CD, Chheang C, Peter K, Jaworowski A, Khoshmanesh K, Baratchi S (2022). Piezo1 response to shear stress is controlled by the components of the extracellular matrix. ACS Appl Mater Interfaces.

[33] Gaub BM, Müller DJ (2017). Mechanical stimulation of Piezo1 receptors depends on extracellular matrix proteins and directionality of force. Nano Lett.

[34] Verkest C, Schaefer I, Nees TA, Wang N, Jegelka JM, Taberner FJ, Lechner SG (2022). Intrinsically disordered intracellular domains control key features of the mechanically-gated ion channel PIEZO2. Nat Commun.

[35] Zhou Z, Martinac B (2023). Mechanisms of PIEZO channel inactivation. Int J Mol Sci.

[36] Wang L, Zhou H, Zhang M, Liu W, Deng T, Zhao Q, Li Y, Lei J, Li X, Xiao B (2019). Structure and mechanogating of the mammalian tactile channel PIEZO2. Nature.

[37] Lin YC, Guo YR, Miyagi A, Levring J, MacKinnon R, Scheuring S (2019). Force-induced conformational changes in PIEZO1. Nature.

[38] Unnithan AR, Sasikala ARK, Shrestha BK, Lincoln A, Thomson T, El Haj AJ (2022). Remotely actuated magnetic nanocarpets for bone tissue engineering: Non-invasive modulation of mechanosensitive ion channels for enhanced osteogenesis. Adv Funct Mater.

[39] Matos AM, Gonçalves AI, Rodrigues MT, Miranda MS, El Haj AJ, Reis RL, Gomes ME (2020). Remote triggering of TGF-β/Smad2/3 signaling in human adipose stem cells laden on magnetic scaffolds synergistically promotes tenogenic commitment. Acta Biomater.

[40] Lee J, Shin W, Lim Y, Kim J, Kim WR, Kim H, Lee JH, Cheon J (2021). Non-contact long-range magnetic stimulation of mechanosensitive ion channels in freely moving animals. Nat Mater.

[41] Del Sol-Fernández S, Martínez-Vicente P, Gomollón-Zueco P, Castro-Hinojosa C, Gutiérrez L, Fratila RM, Moros M (2022). Magnetogenetics: remote activation of cellular functions triggered by magnetic switches. Nanoscale.

[42] Chansoria P, Liu H, Christiansen MG, Schürle-Finke S, Zenobi-Wong M (2023). Untethered: using remote magnetic fields for regenerative medicine. Trends Biotechnol.

[43] Ayoubi R, Ryan J, Biddle MS, Alshafie W, Fotouhi M, Bolivar SG, Moleon VR, Eckmann P, Worrall D, McDowell I, Southern K (2023). Scaling of an antibody validation procedure enables quantification of antibody performance in major research applications. eLife.

[44] Simon-Chica A, Klesen A, Emig R, Chan A, Greiner J, Grün D, Lother A, Rog-Zielinska EA, Ravens U, Kohl P, Schneider-Warme F (2024). Piezo1 stretch-activated channel activity differs between murine bone marrow-derived and cardiac tissue-resident macrophages. J Physiol.

[45] Porebski BT, Balmforth M, Browne G, Riley A, Jamali K, Fürst MJLJ, Velic M, Buchanan A, Minter R, Vaughan T, Holliger P (2024). Rapid discovery of high-affinity antibodies via massively parallel sequencing, ribosome display and affinity screening. Nat Biomed Eng.

[46] Teixeira SPB, Reis RL, Peppas NA, Gomes ME, Domingues RMA (2021). Epitope-imprinted polymers: Design principles of synthetic binding partners for natural biomacromolecules. Sci Adv.

[47] Teixeira SPB, Domingues RMA, Shevchuk M, Gomes ME, Peppas NA, Reis RL (2020). Biomaterials for sequestration of growth factors and modulation of cell behavior. Adv Funct Mater.

[48] Parisi OI, Francomano F, Dattilo M, Patitucci F, Prete S, Amone F, Puoci F (2022). The evolution of molecular recognition: From antibodies to molecularly imprinted polymers (MIPs) as artificial counterpart. J Funct Biomater.

[49] Kang MS, Cho E, Choi HE, Amri C, Lee JH, Kim KS (2023). Molecularly imprinted polymers (MIPs): emerging biomaterials for cancer theragnostic applications. Biomater Res.

[50] Xing R, Ma Y, Wang Y, Wen Y, Liu Z (2019). Specific recognition of proteins and peptides via controllable oriented surface imprinting of boronate affinity-anchored epitopes. Chem Sci.

[51] Medina Rangel PX, Laclef S, Xu J, Panagiotopoulou M, Kovensky J, Tse Sum Bui B, Haupt K (2019). Solid-phase synthesis of molecularly imprinted polymer nanolabels: Affinity tools for cellular bioimaging of glycans. Sci Rep.

[52] Bossi AM, Sharma PS, Montana L, Zoccatelli G, Laub O, Levi R (2012). Fingerprint-imprinted polymer: Rational selection of peptide epitope templates for the determination of proteins by molecularly imprinted polymers. Anal Chem.

[53] Bateman A, Martin MJ, Orchard S, Magrane M, Ahmad S, Alpi E, Bowler-Barnett EH, Britto R, Bye-A-Jee H, Cukura A, Denny P (2023). UniProt: the Universal Protein Knowledgebase in 2023. Nucleic Acids Res.

[54] Jumper J, Evans R, Pritzel A, Green T, Figurnov M, Ronneberger O, Tunyasuvunakool K, Bates R, Potapenko A, Bridgland A, Meyer C (2021). Highly accurate protein structure prediction with AlphaFold. Nature.

[55] Varadi M, Anyango S, Deshpande M, Nair S, Natassia C, Yordanova G, Yuan D, Stroe O, Wood G, Laydon A, Green T (2022). AlphaFold Protein Structure Database: massively expanding the structural coverage of protein-sequence space with high-accuracy models. Nucleic Acids Res.

[56] Douguet D, Honoré E (2019). Mammalian mechanoelectrical transduction: Structure and function of force-gated ion channels. Cell.

[57] Pardo A, Pelaz B, Gallo J, Bañobre-López M, Parak WJ, Barbosa S, del Pino P, Taboada P (2020). Synthesis, characterization, and evaluation of superparamagnetic doped ferrites as potential therapeutic nanotools. Chem Mater.

[58] Bakht SM, Pardo A, Gómez-Florit M, Reis RL, Domingues RMA, Gomes ME (2021). Engineering next-generation bioinks with nanoparticles: moving from reinforcement fillers to multifunctional nanoelements. J Mater Chem B.

[59] Pardo A, Bakht SM, Gomez-Florit M, Rial R, Monteiro RF, Teixeira SPB, Taboada P, Reis RL, Domingues RMA, Gomes ME (2022). Magnetically-assisted 3D bioprinting of anisotropic tissue-mimetic constructs. Adv Funct Mater.

[60] Canfarotta F, Poma A, Guerreiro A, Piletsky S (2016). Solid-phase synthesis of molecularly imprinted nanoparticles. Nat Protoc.

[61] Poma A, Guerreiro A, Whitcombe MJ, Piletska EV, Turner APF, Piletsky SA (2013). Solid-phase synthesis of molecularly imprinted polymer nanoparticles with a reusable template-“plastic antibodies”. Adv Funct Mater.

[62] Ekpenyong-Akiba AE, Canfarotta F, Abd H, Poblocka M, Casulleras M, Castilla-Vallmanya L, Kocsis-Fodor G, Kelly ME, Janus J, Althubiti M, Piletska E (2019). Detecting and targeting senescent cells using molecularly imprinted nanoparticles. Nanoscale Horiz.

[63] Landry JP, Ke Y, Yu GL, Zhu XD (2015). Measuring affinity constants of 1,450 monoclonal antibodies to peptide targets with a microarray-based label-free assay platform. J Immunol Methods.

[64] Canfarotta F, Lezina L, Guerreiro A, Czulak J, Petukhov A, Daks A, Smolinska-Kempisty K, Poma A, Piletsky S, Barlev NA (2018). Specific drug delivery to cancer cells with double-imprinted nanoparticles against epidermal growth factor receptor. Nano Lett.

[65] Wang S, Chennupati R, Kaur H, Iring A, Wettschureck N, Offermanns S (2016). Endothelial cation channel PIEZO1 controls blood pressure by mediating flow-induced ATP release. J Clin Investig.

[66] Li J, Hou B, Tumova S, Muraki K, Bruns A, Ludlow MJ, Sedo A, Hyman AJ, McKeown L, Young RS, Yuldasheva NY (2014). Piezo1 integration of vascular architecture with physiological force. Nature.

[67] Jaeger PK, Passini FS, Niederoest B, Bollhalder M, Fucentese S, Snedeker JG (2025). Matrix and cytoskeletal tension gate stretch-induced calcium signaling. Acta Biomater.

[68] Tomás AR, Gonçalves AI, Paz E, Freitas P, Domingues RMA, Gomes ME (2019). Magneto-mechanical actuation of magnetic responsive fibrous scaffolds boosts tenogenesis of human adipose stem cells. Nanoscale.

[69] Aragona M, Panciera T, Manfrin A, Giulitti S, Michielin F, Elvassore N, Dupont S, Piccolo S (2013). A mechanical checkpoint controls multicellular growth through YAP/TAZ regulation by actin-processing factors. Cell.

[70] Dupont S, Morsut L, Aragona M, Enzo E, Giulitti S, Cordenonsi M, Zanconato F, Le Digabel J, Forcato M, Bicciato S, Elvassore N (2011). Role of YAP/TAZ in mechanotransduction. Nature.

[71] Hasegawa K, Fujii S, Matsumoto S, Tajiri Y, Kikuchi A, Kiyoshima T (2021). YAP signaling induces PIEZO1 to promote oral squamous cell carcinoma cell proliferation. J Pathol.

[72] Liu S, Xu X, Fang Z, Ning Y, Deng B, Pan X, He Y, Yang Z, Huang K, Li J (2021). Piezo1 impairs hepatocellular tumor growth via deregulation of the MAPK-mediated YAP signaling pathway. Cell Calcium.

[73] Zhang T, Li Y, Cheng B, Xu Z, Liu M, Feng J, Bai Y, Yu Y, Jiang P, Geng L, Xu F (2025). Piezo1 activation improves NSCLC liver metastasis immunotherapy by overriding matrix stiffness-mediated bimodal PD-L1/CXCL10 regulation. Adv Sci.

[74] Zheng L, Zhang L, Chen L, Jiang J, Zhou X, Wang M, Fan Y (2018). Static magnetic field regulates proliferation, migration, differentiation and YAP/TAZ activation of human dental pulp stem cells. J Tissue Eng Regen Med.

[75] Zhao F, Zhang L, Wei M, Duan W, Wu S, Kasim V (2022). Mechanosensitive ion channel PIEZO1 signaling in the hall-marks of cancer: Structure and functions. Cancers (Basel).

[76] Zhou W, Liu X, van Wijnbergen JWM, Yuan L, Liu Y, Zhang C, Jia W (2020). Identification of PIEZO1 as a potential prognostic marker in gliomas. Sci Rep.

[77] Parpaite T, Coste B (2017). Piezo channels. Curr Biol.

[78] Kang H, Hong Z, Zhong M, Klomp J, Bayless KJ, Mehta D, Karginov AV, Hu G, Malik AB (2019). Piezo1 mediates angiogenesis through activation of MT1-MMP signaling. Am J Physiol Cell Physiol.

[79] Shinge SAU, Zhang D, Achu Muluh T, Nie Y, Yu F (2021). Mechanosensitive Piezo1 channel evoked-mechanical signals in atherosclerosis. J Inflamm Res.

[80] Apte RS, Chen DS, Ferrara N (2019). VEGF in signaling and disease: Beyond discovery and development. Cell.

[81] Dai R, Wang Z, Samanipour R, Koo K, Kim K (2016). Adipose-derived stem cells for tissue engineering and regenerative medicine applications. Stem Cell Int.

[82] Palumbo P, Lombardi F, Siragusa G, Cifone MG, Cinque B, Giuliani M (2018). Methods of isolation, characterization and expansion of human adipose-derived stem cells (ASCs): An overview. Int J Mol Sci.

[83] Fang F, Liu C, Huang Q, Dong C, Zhang G, Jiang J, Lu S (2024). Effect of static magnetic field on gene expression of human umbilical cord mesenchymal stem cells by transcriptome analysis. Adv Med Sci.

[84] Wu H, Li C, Masood M, Zhang Z, González-Almela E, Castells-Garcia A, Zou G, Xu X, Wang L, Zhao G, Yu S (2022). Static magnetic fields regulate T-Type calcium ion channels and mediate mesenchymal stem cells proliferation. Cells.

[85] Xie Z, Bailey A, Kuleshov MV, Clarke DJB, Evangelista JE, Jenkins SL, Lachmann A, Wojciechowicz ML, Kropiwnicki E, Jagodnik KM, Jeon M (2021). Gene set knowledge discovery with Enrichr. Curr Protoc.

[86] Kuleshov MV, Jones MR, Rouillard AD, Fernandez NF, Duan Q, Wang Z, Koplev S, Jenkins SL, Jagodnik KM, Lachmann A, McDermott MG (2016). Enrichr: a comprehensive gene set enrichment analysis web server 2016 update. Nucleic Acids Res.

[87] Chen EY, Tan CM, Kou Y, Duan Q, Wang Z, Meirelles GV, Clark NR, Ma’ayan A (2013). Enrichr: interactive and collaborative HTML5 gene list enrichment analysis tool. BMC Bioinf.

[88] Dienes B, Bazsó T, Szabó L, Csernoch L (2023). The role of the Piezo1 mechanosensitive channel in the musculoskeletal system. Int J Mol Sci.

[89] Savadipour A, Palmer D, Ely EV, Collins KH, Garcia-Castorena JM, Harissa Z, Kim YS, Oestrich A, Qu F, Rashidi N, Guilak F (2023). The role of PIEZO ion channels in the musculoskeletal system. Am J Physiol Cell Physiol.

[90] Gu Y, Mohammad I, Liu Z (2020). Overview of the STAT-3 signaling pathway in cancer and the development of specific inhibitors (Review). Oncol Lett.

[91] Hu X, Li J, Fu M, Zhao X, Wang W (2021). The JAK/STAT signaling pathway: from bench to clinic. Signal Transduct Targeted Ther.

[92] Widjaja AA, Lim WW, Viswanathan S, Chothani S, Corden B, Dasan CM, Lim R, Singh BK, Tan J, Pua CJ, Lim SY (2024). Inhibition of IL-11 signalling extends mammalian healthspan and lifespan. Nature.

[93] Sheikh E, Tran T, Vranic S, Levy A, Bonfil RD (2022). Role and significance of c-KIT receptor tyrosine kinase in cancer: A review. Bosn J Basic Med Sci.

[94] Sprangers S, Schoenmaker T, Cao Y, Everts V, de Vries TJ (2017). Integrin αMβ2 is differently expressed by subsets of human osteoclast precursors and mediates adhesion of classical monocytes to bone. Exp Cell Res.

[95] Perrone M, Patergnani S, Di Mambro T, Palumbo L, Wieckowski MR, Giorgi C, Pinton P (2023). Calcium homeostasis in the control of mitophagy. Antioxid Redox Signal.

[96] Sukumaran P, Nascimento Da Conceicao V, Sun Y, Ahamad N, Saraiva LR, Selvaraj S, Singh BB (2021). Calcium signaling regulates autophagy and apoptosis. Cells.

[97] Sugimoto A, Miyazaki A, Kawarabayashi K, Shono M, Akazawa Y, Hasegawa T, Ueda-Yamaguchi K, Kitamura T, Yoshizaki K, Fukumoto S, Iwamoto T (2017). Piezo type mechanosensitive ion channel component 1 functions as a regulator of the cell fate determination of mesenchymal stem cells. Sci Rep.

[98] Tadge T, Pattewar A, More N, Babu SS, Velyutham R, Kapusetti G (2024). The role of Piezo1 and Piezo2 proteins in tissue engineering: A Comprehensive review. Eng Regen.

[99] Wang B, Li G, Zhu Q, Liu W, Ke W, Hua W, Zhou Y, Zeng X, Sun X, Wen Z, Yang C (2022). Bone repairment via mechanosensation of Piezo1 using wearable pulsed triboelectric nanogenerator. Small.

[100] Snedeker JG, Gautieri A (2014). The role of collagen crosslinks in ageing and diabetes - the good, the bad, and the ugly. Muscles Ligaments Tendons J.

[101] Ellingson A, Pancheri N, Schiele N (2022). Regulators of collagen crosslinking in developing and adult tendons. Eur Cell Mater.

[102] Lee W, Nims RJ, Savadipour A, Zhang Q, Leddy HA, Liu F, McNulty AL, Chen Y, Guilak F, Liedtke WB (2021). Inflammatory signaling sensitizes Piezo1 mechanotransduction in articular chondrocytes as a pathogenic feed-forward mechanism in osteoarthritis. Proc Natl Acad Sci U S A.

[103] Gao W, Hasan H, Anderson DE, Lee W (2022). The role of mechanically-activated ion channels Piezo1, Piezo2, and TRPV4 in chondrocyte mechanotransduction and mechano-therapeutics for osteoarthritis. Front Cell Dev Biol.

[104] Brylka LJ, Alimy A-R, Tschaffon-Müller MEA, Jiang S, Ballhause TM, Baranowsky A, von Kroge S, Delsmann J, Pawlus E, Eghbalian K, Püschel K (2024). Piezo1 expression in chondrocytes controls endochondral ossification and osteoarthritis development. Bone Res.

[105] Wang X, Tao J, Zhou J, Shu Y, Xu J (2024). Excessive load promotes temporomandibular joint chondrocyte apoptosis via Piezo1/endoplasmic reticulum stress pathway. J Cell Mol Med.

[106] Nims R, Palmer DR, Kassab J, Zhang B, Guilak F (2024). The chondrocyte “mechanome”: Activation of the mechanosensitive ion channels TRPV4 and PIEZO1 drives unique transcriptional signatures. FASEB J.

[107] Huang Z, Huang Y, Ning X, Li H, Li Q, Wu J (2023). The functional effects of Piezo channels in mesenchymal stem cells. Stem Cell Res Ther.

[108] Peng Y, Du J, Günther S, Guo X, Wang S, Schneider A, Zhu L, Braun T (2022). Mechano-signaling via Piezo1 prevents activation and p53-mediated senescence of muscle stem cells. Redox Biol.

[109] He L, Si G, Huang J, Samuel ADT, Perrimon N (2018). Mechanical regulation of stem-cell differentiation by the stretch-activated Piezo channel. Nature.

[110] Ma N, Chen D, Lee J-H, Kuri P, Hernandez EB, Kocan J, Mahmood H, Tichy ED, Rompolas P, Mourkioti F (2022). Piezo1 regulates the regenerative capacity of skeletal muscles via orchestration of stem cell morphological states. Sci Adv.

[111] Zhu H, He W, Ye P, Chen J, Wu X, Mu X, Wu Y, Pang H, Han F, Nie X (2023). Piezo1 in skin wound healing and related diseases: Mechanotransduction and therapeutic implications. Int Immunopharmacol.

[112] Xie Y, Hang L (2024). Mechanical gated ion channel Piezo1: Function, and role in macrophage inflammatory response. Innate Immun.

[113] Choi SH, Aguilar A, Myers J, Kim BG, Eid S, Tomchuck S, Kingsley D, Huang A (2022). Mechanosensory channel Piezo1 is essential in pathogenic T cell-mediated intestinal inflammation. J Immunol.

[114] Syeda R, Florendo MN, Cox CD, Kefauver JM, Santos JS, Martinac B, Patapoutian A (2016). Piezo1 channels are inherently mechanosensitive. Cell Rep.

[115] Jiang W, Del Rosario JS, Botello-Smith W, Zhao S, Lin Y, Zhang H, Lacroix J, Rohacs T, Luo YL (2021). Crowding-induced opening of the mechanosensitive Piezo1 channel in silico. Commun Biol.

[116] Botello-Smith WM, Jiang W, Zhang H, Ozkan AD, Lin YC, Pham CN, Lacroix JJ, Luo Y (2019). A mechanism for the activation of the mechanosensitive Piezo1 channel by the small molecule Yoda1. Nat Commun.

[117] Lüchtefeld I, Pivkin IV, Gardini L, Zare-Eelanjegh E, Gäbelein C, Ihle SJ, Reichmuth AM, Capitanio M, Martinac B, Zambelli T, Vassalli M (2024). Dissecting cell membrane tension dynamics and its effect on Piezo1-mediated cellular mechanosensitivity using force-controlled nanopipettes. Nat Methods.

[118] Sarrió-Ferrández S, Selva E, Taberner FJ (2024). PIEZO1 and PIEZO2 Blade domains are differentially required for channel localization and function. bioRxiv.

[119] Ellefsen KL, Holt JR, Chang AC, Nourse JL, Arulmoli J, Mekhdjian AH, Abuwarda H, Tombola F, Flanagan LA, Dunn AR, Parker I (2019). Myosin-II mediated traction forces evoke localized Piezo1-dependent Ca2+ flickers. Commun Biol.

[120] Sarna NS, Desai SH, Kaufman BG, Curry NM, Hanna AM, King MR (2024). Enhanced and sustained T cell activation in response to fluid shear stress. iScience.

[121] Guo Y, Luo J, Zou H, Liu C, Deng L, Li P (2022). Context-dependent transcriptional regulations of YAP/TAZ. Cancer Lett.

[122] Franklin JM, Ghosh RP, Shi Q, Reddick MP, Liphardt JT (2020). Concerted localization-resets precede YAP-dependent transcription. Nat Commun.

[123] Meyer K, Yserentant K, Cheloor-Kovilakam R, Ruff KM, Chung C-I, Shu X, Huang B, Weiner OD (2025). YAP charge patterning mediates signal integration through transcriptional co-condensates. Nat Commun.

[124] Pan Y, Yoon S, Sun J, Huang Z, Lee C, Allen M, Wu Y, Chang YJ, Sadelain M, Shung KK, Chien S (2018). Mechanogenetics for the remote and noninvasive control of cancer immunotherapy. Proc Natl Acad Sci USA.

[125] Burdick JA, Vunjak-Novakovic G (2009). Engineered microenvironments for controlled stem cell differentiation. Tissue Eng Part A.

[126] Wan X, Liu Z, Li L (2021). Manipulation of stem cells fates: The master and multifaceted roles of biophysical cues of biomaterials. Adv Funct Mater.

[127] Higuchi A, Ling Q-D, Chang Y, Hsu S-T, Umezawa A (2013). Physical cues of biomaterials guide stem cell differentiation fate. Chem Rev.

[128] Oliva MAG, Ciccone G, Fläschner G, Luo J, Voigt JL, Romani P, Genever P, Dobre O, Dupont S, Vassalli M, Roca-Cusachs P (2025). Piezo1 regulates the mechanotransduction of soft matrix viscoelasticity. Nat Commun.

[129] Gomez-Florit M, Labrador-Rached CJ, Domingues RMA, Gomes ME (2022). The tendon microenvironment: Engineered in vitro models to study cellular crosstalk. Adv Drug Deliv Rev.

[130] Monteiro RF, Bakht SM, Gomez-Florit M, Stievani FC, Alves ALG, Reis RL, Gomes ME, Domingues RMA (2023). Writing 3D in vitro models of human tendon within a biomimetic fibrillar support platform. ACS Appl Mater Interfaces.

[131] Ding F, Ma Y, Fan W, Xu J, Pan G (2024). Tailor-made molecular imprints for biological event intervention. Trends Biotechnol.

[132] ExPASy PeptideCutter, can be found under (2021). https://web.expasy.org/peptide_cutter/.

[133] Gasteiger E, Hoogland C, Gattiker A, Duvaud S, Wilkins MR, Appel RD, Bairoch A, Walker John M (2005). The Proteomics Protocols Handbook, Springer Protocols Handbooks.

[134] U. S. National Library of Medicine (2021). National Center for Biotechnology Information. BLASTP, can be found under.

[135] Teixeira SPB, Pardo A, Bakht SM, Gomez-Florit M, Reis RL, Gomes ME, Domingues RMA (2024). Guiding stem cell tenogenesis by modulation of growth factor signaling and cell-scale biophysical cues in bioengineered constructs. Adv Funct Mater.

[136] Carvalho PP, Wu X, Yu G, Dias IR, Gomes ME, Reis RL, Gimble JM (2011). The effect of storage time on adipose-derived stem cell recovery from human lipoaspirates. Cells Tissues Organs.

[137] Gemperle J, Liße D, Kappen M, Secret E, Coppey M, Gregor M, Menager C, Piehler J, Caswell P (2025). Live-cell magnetic manipulation of recycling endosomes reveals their direct effect on actin protrusions to promote invasive migration. Sci Adv.

[138] Meeker DC (2018). Finite element method magnetics (FEMM) 4.2 (28Feb2018 build), can be found under.

[139] Toni LS, Garcia AM, Jeffrey DA, Jiang X, Stauffer BL, Miyamoto SD, Sucharov CC (2018). Optimization of phenol-chloroform RNA extraction. MethodsX.

[140] Livak KJ, Schmittgen TD (2001). Analysis of relative gene expression data using real-time quantitative PCR and the 2 −ΔΔCT method. Methods.

[141] Hatakeyama M, Opitz L, Russo G, Qi W, Schlapbach R, Rehrauer H (2016). SUSHI: an exquisite recipe for fully documented, reproducible and reusable NGS data analysis. BMC Bioinf.

[142] Wingett SW, Andrews S (2018). FastQ Screen: A tool for multi-genome mapping and quality control. F1000Res.

[143] Dobin A, Davis CA, Schlesinger F, Drenkow J, Zaleski C, Jha S, Batut P, Chaisson M, Gingeras TR (2013). STAR: Ultrafast universal RNA-seq aligner. Bioinformatics.

[144] Bray NL, Pimentel H, Melsted P, Pachter L (2016). Near-optimal probabilistic RNA-seq quantification. Nat Biotechnol.

[145] Metsalu T, Vilo J (2015). ClustVis: A web tool for visualizing clustering of multivariate data using Principal Component Analysis and heatmap. Nucleic Acids Res.

[146] Akhmedov M, Martinelli A, Geiger R, Kwee I (2020). Omics Playground: A comprehensive self-service platform for visualization, analytics and exploration of Big Omics Data. NAR Gemon Bioinform.

[147] Goedhart J, Luijsterburg MS (2020). VolcaNoseR is a web app for creating, exploring, labeling and sharing volcano plots. Sci Rep.

[148] Korotkevich G, Sukhov V, Budin N, Shpak B, Artyomov MN, Sergushichev A (2021). Fast gene set enrichment analysis. bioRxiv.

