# Supplementary material for: Submolecular modulation of PIEZO1 mechanotransduction with wireless tailor-made nanoswitches
Source: Bioact Mater. Author manuscript; Available in PMC 2026 Sep 1. (PMC13137217; doi:10.1016/j.bioactmat.2026.03.051)
Supplement: Supplementary file 1 [file EMS213671-supplement-Supplementary_data.docx]

Supporting Information

Submolecular Modulation of PIEZO1 Mechanotransduction with Wireless Tailor-made Nanoswitches

The present document contains Table S1 through S9 and Figure S1 through S7, as well as further details on the Materials and Methods.

**Table S1**. Human PIEZO1 peptide sequences (8-15 amino acids length only) resulting from ExPASy Peptide Cutter [1,2] processing ordered by BLAST-P maximum score and E(rror) value [3].

| Position | Sequence | Length (a.a.) | Molecular weight (Da) | Max score | Total score | E value |
| --- | --- | --- | --- | --- | --- | --- |
| 2070 | MFNQNVVAQLWYFVK | 15 | 1887,229 | 56,2 | 84,6 | 6E-12 |
| 1562 | YLLTQELLQGGEVHR | 15 | 1755,991 | 51,1 | 92,1 | 3E-10 |
| 365 | ELELAELDQWPQER | 14 | 1755,902 | 50,7 | 128 | 4E-10 |
| **2426** | **TDCNLLPMVIFSDK** | **14** | **1595,889** | **50,3** | **88,7** | **6E-10** |
| 2386 | QLQPNEEADYLGVR | 14 | 1631,762 | 48,6 | 61,5 | 2E-09 |
| 1797 | YDLVQLMALFFHR | 13 | 1652,975 | 48,1 | 88,2 | 3E-09 |
| 1152 | GEPNPVPNFIHCR | 13 | 1479,676 | 47,7 | 47,7 | 4E-09 |
| 2110 | YNHLNLFLFQGFR | 13 | 1668,918 | 47,7 | 59,4 | 4E-09 |
| 801 | LLELAAGFSDVLSR | 14 | 1490,72 | 44,8 | 113 | 5E-08 |
| 2082 | CIYFALSAYQIR | 12 | 1447,715 | 43,9 | 43,9 | 7E-08 |
| **920** | **GPVDPANWFGVR** | **12** | **1314,466** | **43,1** | **64,7** | **1E-07** |
| 573 | TQTLLQSLGELVK | 13 | 1429,677 | 42,2 | 82,7 | 3E-07 |
| 1631 | SGSEEAVTDPGER | 13 | 1333,331 | 42,2 | 71,9 | 3E-07 |
| 828 | LVALYTVWVALK | 12 | 1375,717 | 41,8 | 54,7 | 4E-07 |
| 984 | QQLDQDLLGCLK | 12 | 1373,587 | 41,8 | 54,3 | 4E-07 |
| 2302 | ELYNGTADITLR | 12 | 1365,506 | 41,4 | 95,9 | 6E-07 |
| 201 | VTAHWLLVAAGR | 12 | 1293,534 | 40,9 | 107 | 8E-07 |
| 1311 | VFLSHYYLHVR | 11 | 1433,676 | 40,9 | 85,1 | 6E-07 |
| 1643 | EAGASLYQGLMR | 12 | 1295,476 | 40,9 | 65,1 | 8E-07 |
| 2335 | HMLALAPNSTAR | 12 | 1281,495 | 40,9 | 100 | 8E-07 |
| 1772 | YENKPYFPPR | 10 | 1310,475 | 38,8 | 38,8 | 3E-06 |
| 2502 | ELELEEELYAK | 11 | 1365,5 | 38,8 | 92,9 | 4E-06 |
| 1323 | ADLQATALLASR | 12 | 1229,399 | 38 | 81,9 | 9E-06 |
| 97 | LDQLLGPSCSR | 11 | 1188,364 | 37,5 | 53,4 | 1E-05 |
| 1334 | GFALYNAANLK | 11 | 1181,357 | 37,1 | 48,8 | 1E-05 |
| 2372 | APNGPEANPVK | 11 | 1093,205 | 37,1 | 37,1 | 1E-05 |
| 993 | YFINFFFYK | 9 | 1288,511 | 36,7 | 47,5 | 1E-05 |
| 1092 | WLYLPDFFR | 9 | 1256,469 | 36,7 | 51,8 | 1E-05 |
| 738 | QDAVSGTPLLR | 11 | 1156,304 | 36,3 | 50,1 | 3E-05 |
| 1544 | HHGTMSDVLR | 10 | 1152,293 | 36,3 | 62,1 | 2E-05 |
| 1083 | AVPMNSALIK | 10 | 1043,29 | 34,6 | 34,6 | 1E-04 |
| 1381 | SRPQDTLGPK | 10 | 1098,224 | 34,1 | 77,6 | 1E-04 |
| 1953 | FFHDILHTK | 9 | 1157,337 | 34,1 | 43,7 | 1E-04 |
| 2323 | GGTVEYANEK | 10 | 1067,12 | 34,1 | 34,1 | 1E-04 |
| 2488 | LCQDIFLVR | 9 | 1106,348 | 33,7 | 33,7 | 2E-04 |
| 1894 | GAAAIEAEDR | 10 | 1002,049 | 32,9 | 32,9 | 4E-04 |
| 1903 | EEEEGEEEK | 9 | 1107,05 | 32,5 | 176 | 4E-04 |
| 2119 | LVPFLVELR | 9 | 1085,355 | 32 | 61,7 | 6E-04 |
| 58 | CGLQGHTGR | 9 | 928,034 | 31,6 | 31,6 | 9E-04 |
| 1652 | TASELLLDR | 9 | 1017,147 | 30,3 | 54,5 | 3E-03 |
| 1470 | QQEQEQAR | 8 | 1016,035 | 29,5 | 47,1 | 4E-03 |
| 816 | LLELHVFK | 8 | 998,233 | 29,1 | 50,7 | 6E-03 |
| 1134 | MAGVNTDR | 8 | 862,956 | 29,1 | 29,1 | 6E-03 |
| 341 | AYRPSGQR | 8 | 934,023 | 28,6 | 28,6 | 8E-03 |
| 510 | QLGLEHTR | 8 | 953,066 | 28,6 | 59,6 | 8E-03 |
| 2096 | ILGNFLTK | 8 | 905,105 | 28,2 | 28,2 | 1E-02 |

Color legend:

**Selected sequences**

Intracellular beam region (1300-1361) and connected intracellular region (1362-1690)

Transmembrane helix

C-terminal extracellular domain/Cap-structure (2214-2457)

Extracellular loop L19-20 (870-921)

**Figure S1**. Localization of highest scoring sequences (highlighted in green) that were not immediately excluded in the human PIEZO1 structure (UniProt [4] entry Q92508), showing their transmembrane or intracellular placement. Adapted from AlphaFold Protein Structure Database under Creative Commons Attribution 4.0 (CC-BY 4.0) license terms [5,6].

**Table S2.** Size and surface charge distribution of the different nanoparticles synthesized in this study.

| Sample | Hydrodynamic diameter (nm) | Polydispersity index (PDI) | Zeta potential (mV) |
| --- | --- | --- | --- |
| SPIONs | 27.7 ± 1.5 | 0.216 ± 0.013 | -42.4 ± 2.7 |
| BINPs | 212 ± 18 | 0.607 ± 0.092  **  ** | -22.8 ± 0.8 |
| LooPINS | 248 ± 37 | 0.578 ± 0.103 | -25.4 ± 1.8 |
| CaPINS | 234 ± 16 | 0.390 ± 0.044 | -24.8 ± 1.1  * |

Statistical analysis performed by one-way ANOVA, followed by Tukey’s t-test for individual comparisons between BINPs, LooPINS, and CaPINS (n=5): * p=0.041; ** p<0.01.

**Table S3**. Mass values of SPIONs and monomers used in each MINP synthesis, and saturation magnetization values (M_S_) from magnetometry measurements using a superconducting quantum interference device (SQUID).

| Sample | Mass (mg) | M_S_ (emu·g^-1­^) |
| --- | --- | --- |
| SPIONs | 1.00 | 65.0 |
| CaPINS | 82.1 | 0.740 |
| Ratio (%) | 1.22 | 1.14 |

**Table S4**. Surface plasmon resonance (SPR) fitting results from single cycle kinetic (SCK) assays.

| Sample | *k_a_* (M^-1^ s^-1^) | SE(*k_a_*) | | *k_d_* (s^-1^) | SE(*k_d_*) | | *K_D_* (M) | *R_max_* (RU) | SE(*R_max_*) | | Chi^2^ (RU^2^) | U-value |
| --- | --- | --- | --- | --- | --- | --- | --- | --- | --- | --- | --- | --- |
|  |  | (M^-1^ s^-1^) | (%) |  | (s^-1^) | (%) |  |  | (RU) | (%) |  |  |
| LooPINS | 4.8×10^5^ | 4.2×10^3^ | 0.87 | 2.5×10^-4^ | 6.4×10^-6^ | 2.6 | 5.2×10^-10^ | 108 | 0.61 | 0.57 | 4.86 | 3 |
|  | 2.4×10^6^ | 3.7×10^4^ | 1.5 | 6.7×10^-4^ | 2.0×10^-5^ | 3.0 | 2.7×10^-10^ | 24.0 | 0.18 | 0.75 | 3.07 | 5 |
|  | 1.6×10^6^ | 3.9×10^4^ | 2.4 | 4.5×10^-4^ | 2.8×10^-5^ | 6.2 | 2.8×10^-10^ | 18.2 | 0.25 | 1.4 | 3.51 | 9 |
| CaPINS | 3.5×10^5^ | 1.0×10^4^ | 2.9 | 1.6×10^-3^ | 4.5×10^-5^ | 2.7 | 4.7×10^-9^ | 40.6 | 0.52 | 1.3 | 26.2 | 7 |
|  | 3.9×10^6^ | 1.9×10^5^ | 4.8 | 1.1×10^-3^ | 6.7×10^-5^ | 6.2 | 2.7×10^-10^ | 27.6 | 0.57 | 2.1 | 34.9 | 12 |
|  | 6.9×10^5^ | 2.7×10^4^ | 3.9 | 1.1×10^-3^ | 4.5×10^-5^ | 4.2 | 1.5×10^-9^ | 37.4 | 0.82 | 2.2 | 23.4 | 9 |

U-values below 25 indicate that absolute values for two or more of the parameters (rate constants and R_max_) are not correlated and could be determined independently. *k_a_* – association rate; *k_d_* – dissociation rate; SE – standard error; *K_D_* – equilibrium dissociation constant; *R_max_* – maximum response; RU – response units.

**Figure S2**. Confocal microscopy visualization of a droplet of fluorescein-labelled nanoswitches suspension (green). Dashed line marks the droplet edge. Scale bar: 250 μm.

**Figure S3**. Simulation of combined magnetic field produced by the tower of permanent magnets used as magnetic probe for PIEZO1 actuation through PINS. Performed with Ansys Maxwell 3D software.

**Figure S4.** Setup for magnetic stimulation of cells cultured with PIEZO1 nanoswitches. **A**) Photographs of Magnefect Nano II magnetic stimulation equipment, showing (i) the overall constitution of the system and its several components and (ii) the positioning of culture plates over the magnet array. **B**) Schematic representation of a transversal cut of a 24-well culture plate on top of the oscillating magnet array, with the respective dimensions used for magnetic simulations. **C**) Finite Element Method Magnetics (FEMM) simulation results for the magnetic flux density (|B| , T) applied on cells during the culture period. **D**) FEMM calculation of |B| along i) the distance from the center of each cell culture surface and ii) the diameter of each well, at 265 µm from the surface (255 µm coverslip thickness + 10 µm cell thickness). The dotted line represents the average value of |B| at this distance.

**Figure S5**. Cell viability measured by resorufin absorbance at 570 nm normalized by resazurin absorbance at 600 nm on day 7 of culture with LooPINS or CaPINS.

[LooPINS] = [CaPINS] = 0.0228 g L^-1^ (concentration used in magnetic stimulation experiments); [LooPINS (2x)] = [CaPINS (2x)] = 0.0456 g L^-1^. 100 % level was set to the average of the No PINS group. Data presented as median ± IQR along with individual values (n=3 biological replicates; 3 technical replicates). Statistical analysis by one-way Analysis of Variance (ANOVA), followed by Tukey’s t-test for individual comparisons.

**Table S5.** Statistical analysis by Kruskal-Wallis test followed by Dunn’s test for individual comparisons (α=0.05) between the distributions of the maximum fluorescence intensity per cell measured in TIRF microscopy images (ΔF_MAX_/F_0_) (Figure 4 A iii).

| Samples | Significance | Adjusted P value |
| --- | --- | --- |
| BINPs (+GsMTx4) vs. LooPINS (+Mag) | **** | <0.0001 |
| BINPs (+GsMTx4) vs. CaPINS (+Mag) | **** | <0.0001 |
| BINPs (+Mag) vs. LooPINS (+GsMTx4) | **** | <0.0001 |
| BINPs (+Mag) vs. CaPINS (+GsMTx4) | **** | <0.0001 |
| BINPs (+Mag) vs. No NPs (+Mag) | ** | 0.0024 |
| BINPs (+Mag) vs. LooPINS (+Mag) | **** | <0.0001 |
| BINPs (+Mag) vs. CaPINS (-) | ** | 0.0034 |
| BINPs (+Mag) vs. CaPINS (+Mag) | **** | <0.0001 |
| LooPINS (+GsMTx4) vs. LooPINS (+Mag) | **** | <0.0001 |
| LooPINS (+GsMTx4) vs. CaPINS (+Mag) | **** | <0.0001 |
| CaPINS (+GsMTx4) vs. No NPs (+GsMTx4) | ** | 0.0076 |
| CaPINS (+GsMTx4) vs. LooPINS (+Mag) | *** | 0.0001 |
| CaPINS (+GsMTx4) vs. CaPINS (+Mag) | **** | <0.0001 |
| No NPs (+GsMTx4) vs. LooPINS (+Mag) | **** | <0.0001 |
| No NPs (+GsMTx4) vs. CaPINS (+Mag) | **** | <0.0001 |
| No NPs (+Mag) vs. LooPINS (+Mag) | * | 0.0135 |
| No NPs (+Mag) vs. CaPINS (+Mag) | ** | 0.0084 |
| LooPINS (-) vs. LooPINS (+Mag) | ** | 0.0082 |
| LooPINS (-) vs. CaPINS (+Mag) | ** | 0.0054 |
| LooPINS (+Mag) vs. CaPINS (-) | ** | 0.0029 |
| CaPINS (-) vs. CaPINS (+Mag) | ** | 0.0015 |

Only statistically significant comparisons (p<0.05) are noted in this table.

**Table S6**. Statistical analysis by one-way Analysis of Variance (ANOVA) followed by Tukey’s post-hoc tests for individual comparisons between nuclear-to-cytoplasm YAP fluorescence intensity ratios (Figure 4 B).

| Samples | Significance | Adjusted P value |
| --- | --- | --- |
| BINP+Mag vs. CaPINS | ns | 0.7722 |
| BINP+Mag vs. CaPINS+Mag | * | 0.0399 |
| BINP+Mag vs. LooPINS | ns | 0.1926 |
| BINP+Mag vs. LooPINS + Mag | * | 0.0352 |
| CaPINS vs. CaPINS +Mag | ** | 0.0016 |
| CaPINS vs. LooPINS | ns | 0.7737 |
| CaPINS vs. LooPINS + Mag | *** | 0.0008 |
| CaPINS+Mag vs. LooPINS | *** | 0.0001 |
| CaPINS+Mag vs. LooPINS + Mag | ns | 0.9984 |
| LooPINS vs. LooPINS + Mag | **** | <0.0001 |
|  |  |  |
|  |  |  |

**Figure S6.** Expression of select genes downstream of PIEZO1 activity in ECs after 1 day of culture. Quantitative real-time polymerase chain reaction (qPCR) results are normalized to transcript levels of *GAPDH* (glyceraldehyde 3-phosphate dehydrogenase) and displayed as fold changes relative to gene expression in day 0 (incubation with nanoswitches) (n=3 biological replicates; 2 technical replicates). Statistical analysis by two-way ANOVA followed by Tukey’s post hoc tests for individual comparisons between experimental groups at the same time point. The dotted line represents the basal expression for each marker (“1-fold”) on experimental day 0. Complete data for statistical analysis are displayed in **Table S7** below. *PIEZO1* – Piezo Type Mechanosensitive Ion Channel Component 1; *YAP* – Yes1 associated transcriptional regulator; *VEGFA* – vascular endothelial growth factor A.

**Table S7.** Statistical analysis by 2-way ANOVA followed by Tukey’s post-hoc tests for individual comparisons between samples of quantitative real-time polymerase chain reaction (qPCR) results in endothelial cells on day 1 (Figure S6).

| Gene | Samples | Significance level | Adjusted P value |
| --- | --- | --- | --- |
| *VEGF* | BINPs vs. CaPINS + Mag | ** | 0.0036 |
|  | LooPINS vs. CaPINS + Mag | * | 0.0169 |
| *YAP* | BINPs vs. CaPINS + Mag | * | 0.0105 |
|  | LooPINS vs. CaPINS + Mag | * | 0.0295 |
|  | LooPINS + Mag vs. CaPINS + Mag | * | 0.0394 |
| *PIEZO1* | BINPs vs. CaPINS | * | 0.0175 |
|  | BINPs vs. CaPINS + Mag | **** | <0.0001 |
|  | LooPINS vs. CaPINS | * | 0.0221 |
|  | LooPINS vs. CaPINS + Mag | **** | <0.0001 |
|  | CaPINS vs. LooPINS + Mag | * | 0.0103 |
|  | CaPINS vs. CaPINS + Mag | ** | 0.0080 |
|  | BINPs+Mag vs. CaPINS + Mag | **** | <0.0001 |
|  | LooPINS + Mag vs. CaPINS + Mag | **** | <0.0001 |

Only statistically significant comparisons (p<0.05) are noted in this table.

**Table S8.** Statistical analysis by 2-way ANOVA followed by Tukey’s post-hoc tests for individual comparisons between samples of qPCR results in endothelial cells with or without *PIEZO1* transcript knockdown on day 7 (Figure 4 C).

| Gene | Samples | Significance level | Adjusted P value |
| --- | --- | --- | --- |
| *VEGF* | BINPs vs. CaPINS + Mag | ** | 0.0036 |
|  | LooPINS vs. CaPINS + Mag | * | 0.0169 |
| *YAP* | BINPs vs. CaPINS + Mag | * | 0.0105 |
|  | LooPINS vs. CaPINS + Mag | * | 0.0295 |
|  | LooPINS + Mag vs. CaPINS + Mag | * | 0.0394 |
| *PIEZO1* | BINPs vs. CaPINS | * | 0.0175 |
|  | BINPs vs. CaPINS + Mag | **** | <0.0001 |
|  | LooPINS vs. CaPINS | * | 0.0221 |
|  | LooPINS vs. CaPINS + Mag | **** | <0.0001 |
|  | CaPINS vs. LooPINS + Mag | * | 0.0103 |
|  | CaPINS vs. CaPINS + Mag | ** | 0.0080 |
|  | BINPs+Mag vs. CaPINS + Mag | **** | <0.0001 |
|  | LooPINS + Mag vs. CaPINS + Mag | **** | <0.0001 |

**Figure S7.** Transcriptomic analysis of human adipose tissue-derived stem cells (hASCs) under (+Mag) or without (no Mag) magnetomechanical actuation through biotin-imprinted nanoparticles (BINPs). **Left,** heatmap of gene-level hierarchical clustering of the 31 differentially expressed genes (DEG). Columns represent individual samples (n=3). Blue denotes downregulated genes; orange denotes upregulated genes. **Right**, volcano plot of DEG. Colored dots show significantly expressed genes, as determined by DESeq2 methods, with the horizontal line corresponding to a false discovery rate (FDR) < 0.05 and vertical lines are at a cutoff of log_2_[Fold change] ± 1.

**Table S9**. List of primers used in qPCR studies.

| Target | NCBI reference | Primers | Sequence 5' -3' |
| --- | --- | --- | --- |
| *GAPDH* | NM_002046.5 | Forward | GAT TTG GTC GTA TTG GGC GC |
| *VEGFA* | NM_003376.6 | Forward | GAC AGA TCA CAG GTA CAG GG |
|  |  | Reverse | AGA AGC AGG TGA GAG TAA GC |
| *PIEZO1* | NM_001142864.4 | Forward | ACT TTC CCA TCA GCA CTC GG |
|  |  | Reverse | CCA CGA AGT CCT TGA GAC CC |
| *YAP* | NM_006106.5 | Forward | ACT GCT TCG GCA GGA GTT AG |
|  |  | Reverse | GAG GGA CAC TGT AGC TGC TC |

*GAPDH* - glyceraldehyde-3-phosphate dehydrogenase; *VEGFA* – vascular endothelial growth factor A; *PIEZO1* – piezo type mechanosensitive ion channel component 1; *YAP* - Yes1 associated transcriptional regulator.

**Materials and Methods (Supplementary)**

*Solid phase molecular imprinting of nanoparticles (MINPs)* *– glass bead derivatization with template molecule:* Protocol followed for synthesis of MINPs was adapted from Ref. [7]. First, glass bead surface was activated by boiling in 1 M NaOH (PanReac AppliChem, Spain, 0.8 mL of solution per g of glass beads) for 15 min., and then thoroughly rinsing with deionized water (eight times with 3.33 mL/g beads). PBS was used to neutralize the base (5 mL/g beads) followed by repeated rinsing with deionized water to remove potential salt residues (until pH was ~7). Beads were rinsed twice with acetone (3.33 mL/g beads) and dried at 80 °C for 3 h. Afterward, dry beads were incubated in a freshly prepared solution of either (3‑aminopropyl) trimethoxysilane or (3‑glycidyloxypropyl) trimethoxysilane 2 % (v/v) in anhydrous toluene (Carlo Erba, France) for 24 h at room temperature (RT) (0.4 mL/g beads). Beads were not stirred but simply hand swirled gently from time to time, as collisions between beads abrade the surface and lead to the formation of glass dust. Then, they were decanted onto a sintered disc filter funnel, rinsed with at least eight volumes of acetone and one volume of methanol, and left to dry under vacuum. For biotin immobilization, amine-functionalized beads were incubated overnight in PBS pH 7.4 (0.4 mL/g beads) containing 0.5 g·L^-1^ biotin, 10 g·L^-1^ N-(3-dimethylaminopropyl)-N′-ethylcarbodiimide hydrochloride (EDC), and 15 g·L^-1^ N-hydroxysuccinimide (NHS). For PIEZO1 epitope immobilization, epoxy-functionalized beads were incubated overnight in 10 mM carbonate buffer pH 9.0 (1 mL/g beads) containing 0.2 g·L^-1^ epitope (Cap epitope: TDCNLLPMVIFSDK, Loop 19-20 epitope: K-GPVDPANWFGVR, custom production by GeneCust, France). The resulting epitope-beads were then washed several times with carbonate buffer (8 x 8 mL/g beads). Unreacted epoxide groups were blocked with 1 M ethanolamine pH 9.0 (3 mL/g beads) for 2 h followed by washing with PBS (8 x 8 mL/g beads). Finally, beads were rinsed with deionized water (15 volumes) on a sintered disc filter funnel, under vacuum. Confirmation of bead functionalization was performed by fluorescein labeling as previously described [8].

*Solid phase imprinting of MINPs – polymerization*: *N*-isopropylacrylamide (3.4 mM), N,N′-methylenebisacrylamide (0.13 mM), *N*-*tert*-butylacrylamide (2.6 mM), acrylic acid (0.32 mM) and *N*‑(3‑aminopropyl)methacrylamide hydrochloride (0.33 mM) were dissolved in UP water. 2 mg of hydrophilic Zn-SPIONs were added to the solution and thoroughly mixed. To produce fluorescent MINPs for microscopy visualization, fluorescein *O*-methacrylate (0.13 mM) was added to the previous solution. The solution was degassed by sonicating under vacuum using a Sonics VCX-130PB-220 (Sonics & Materials, USA) with 13 mm probe for 15 min. followed by purging with N_2_ for 30 min. via a long needle. Template-derivatized glass beads were degassed by performing three cycles of alternate vacuum/N_2_. The degassed solution of monomers was poured onto the glass beads while gently hand swirling, and flushing of the flask with N_2_ was continued for 5-10 min. Next, ammonium persulfate (0.26 M) and tetramethylethylenediamine (0.66 M) in UP water was added through a long needle into the bulk of the mixture while swirling gently to homogenize. The mixture was left to react for 2 h at RT under N_2_ flow, periodically swirling it gently. After synthesis, the mixture was transferred to a solid phase extraction tube fitted with a 20-μm porosity polyethylene frit. The tube was fitted to a filtering flask, the solution was removed by vacuum filtration and then replaced with 30 mL of fresh deionized water at RT. Unreacted monomers and low-affinity polymers were removed by repeating this process for 9 times. Then, the outlet of the tube was closed, 30 mL of UP water (prewarmed at 70 °C) were added to the glass beads, and the tube was placed in a water bath at 70 °C for 15 min. After incubation, MINPs were collected by applying vacuum. This incubation step was repeated 3 times, for 2 min. each. The collected MINP suspension was concentrated by ultrafiltration using Vivaspin 20 Ultrafiltration Unit (30 kDa molecular weight cutoff; Sartorius AG, Germany), performing consecutive centrifugations at 15 °C for 15 min. at 3200 × g, until reducing it to the final desired volume. The resulting suspension was then freeze-dried, and the yield was calculated by weighing the dry nanoparticles.

*Determination of MINP affinity for template epitope by SPR*: Biacore X100 (Cytiva, Sweden) was used throughout the following procedures. All solutions were filtered through a 0.22 µm filter before being used with Biacore X100. Sensor chip CM5 (Cytiva, Sweden) was used to immobilize PIEZO1 epitopes. First, the best pH was scouted to maximize ligand immobilization.

Piezo1 Cap epitope was diluted to 2.5 mg·mL^-1^ in 10 mM acetate buffer at pH 3.5, 4.0, 4.5, 5.0, and 5.5. Biacore Wizard Template was followed for pH scouting procedure in flow cell no. 2 (Fc2), using PBS-P+ buffer 1x (Cytiva, Sweden) as running buffer. Regeneration buffer was composed of 200 mM sodium hydroxide and 1 M sodium chloride in UP water. Since maximal response was achieved at pH 4.5, immobilization was performed on Fc2 using Biacore Wizard template parameters: i) flow rate: 5 µL·min^-1^, ii) contact time: 500 s; iii) ligand: 2.5 mg·mL^-1^ Piezo1 Cap epitope in pH 4.5 acetate buffer. Blank immobilization was performed on the reference Fc1, by loading it with a mixture of EDC (75 g·L^-1^; Cytiva) and NHS (11.5 g·L^-1^; Cytiva), followed by ethanolamine HCl pH 8.5 (1.0 M, Cytiva) to block reactive groups.. Finally, regeneration buffer was injected 5 times for 30 s onto the chip surface to remove loosely bound molecules.

For pH scouting, PIEZO1 Loop 19-20 epitope was diluted to 5 mg·mL^-1^ in 10 mM acetate buffer at pH 5, 5.5, 6, 6.5, 7, 8, and 9. The remaining procedure for pH scouting was similar to the one used for the Cap epitope but using HBS-EP+ as running buffer instead. Maximal response was attained at pH 6, which was then used for immobilization onto Fc2 in a CM5 Sensor Chip: i) flow rate: 5 µL·min^-1^, ii) contact time: 600 s, iii) ligand: 5 mg·mL^-1^ PIEZO1 Loop 19-20 epitope in pH 6 acetate buffer..

To assess MINP interaction with the template epitope, Biacore single cycle kinetics standard procedure was followed, consisting in five successive injections at increasing concentrations onto the sensor chip. Apparent MINP molarities ([*MINPs*], mol·L^-1^) were calculated according to Equation 1 [9],

$\left[ MINPs \right]=\frac{6}{\pi N_{A}d^{3}\rho}X$	(1)

where *N_A_* is Avogadro’s number (mol^-1^), *d* is the hydrodynamic diameter of particles (cm), *ρ* is the polymer density (g·cm^-3^), and *X* is polymer weight concentration (g·L^-1^). The *ρ* value was assumed as the estimated by Ogawa *et al.* for *N*‑isopropylacrylamide-based swollen particles, being approx. 0.01 g·cm^‑3^ [10]. A start-up cycle was performed to stabilize the sensor chip surface, where running buffer was injected five consecutive times. Two blank cycles were then performed using the same settings as the assay proper, but injecting running buffer instead of MINP samples. Finally, MINPs were injected at increasing concentrations for 120 s each, with 600 s of dissociation time after the last injection. A single injection of regeneration buffer for 120 s was performed to clean the sensor surface at the end of the run. The resulting sensorgram was analyzed using Biacore X100 Evaluation Software™. Reference curves (Fc1) were automatically subtracted from each active sensorgram. The average of the two blank cycles was then also subtracted from the test run sensorgram. The subtracted curves were finally fit using 1:1 binding kinetics model. Three replicates were performed for each MINP type.

*Magnetic field simulations:* Finite Element Method Magnetics (FEMM) Version 4.2 (28Feb2018 Build) was applied to simulate the magnetic field produced by the Magnefect-LT device used in this study [11]. First, in the Problem Definition, Problem Type was set to ‘Planar’ since the simulation is performed in a 2D transversal cut across the center of a row of wells. The remaining parameters of Problem Type were accepted as automatically filled in by the software. To represent a row of six magnets from the Magnefect-LT 24-well array, a series of six 14 x 8 mm blocks was drawn, with 2 mm interspacing. Their properties were defined by setting Block Type to ‘N42’ at alternating magnetic field directions. A 98 x 3 mm depiction of a transversal slice of a culture plate was drawn on top, with six 14 x 2 mm wells coinciding with each magnet (thickness of the wells was considered 1 mm, according to plate specifications). An open boundary was built with 7 layers and a radius of 77 mm, with the magnet block at its center. This construction is depicted in Figure S4 B. The mesh was generated using standard parameters, and results were then analyzed.

Figure S4 C depicts the generated magnetic flux density map. The magnitude of the magnetic flux density (|B|) was plotted:

a) longitudinally over a 3 mm line running perpendicular to the center of a culture well, starting from its surface (Figure S4 D.i, Supporting Information);

b) along a line parallel to the diameter of a culture well 265 µm away from its surface (Figure S4 D.ii, Supporting Information); this was an estimated average location of MINPs, accounting for the thickness of a coverslip placed within the well (255 um), plus the average thickness of a spread endothelial cell (10 um). The dashed line in Figure S4 D.ii marks the average magnetic field which should act on MINPs.

*Calculation of translational magnetic force applied on nanoswitches*: For a magnetic particle in a magnetic field,^[14][1]^ the force that develops, ***F_M_*** (N = kg·m·s^‑2^), is given by Equation 2

$\boldsymbol{F}_{\boldsymbol{M}}\boldsymbol{=\mu\cdot}\boldsymbol{\nabla}\boldsymbol{B}$	(2)

where ***µ*** is the magnetic moment (A·m^2^) and ***B*** is the magnetic flux density/magnetic induction (T = kg·s^‑2^·A^‑1^), with **∇*B*** being the gradient of *B* (T·m^‑1^ = kg·s^‑2^·A^‑1^·m^‑1^).

The magnetic moment (emu = 10^-3^ A·m^2^) of MINP samples was determined by magnetometry measurements, which were normalized by sample mass, resulting in the mass magnetization of MINPs, henceforth represented by ***M*** (emu·g^‑1^ = 10^‑3^ A·m^2^ (10^‑3^ kg)^‑1^ = A·m^2^·kg^‑1^). This means that the magnetic moment of a single MINP is given by Equation 3

$\boldsymbol{\mu=M\cdot}\boldsymbol{m}_{\boldsymbol{MINP}}$	(3)

*m_MINP_* being the mass of a single MINP (kg). To estimate this value, the relationship between density, mass, and volume was used (Equation 4)

$\boldsymbol{m}_{\boldsymbol{MINP}}\boldsymbol{=}\boldsymbol{\rho}_{\boldsymbol{MINP}}\boldsymbol{\cdot}\boldsymbol{v}_{\boldsymbol{MINP}}$	(4)

The density of MINPs (***ρ_MINP_***, g·cm^-3^) was approximated by factoring the densities of its main constituent elements, poly(*N*-isopropylacrylamide) (PNIPAAm) and iron oxide nanoparticles (SPIONs), according to the mass fraction of each, as per Equation 5

$\boldsymbol{\rho}_{\boldsymbol{MINP}}\boldsymbol{=}\boldsymbol{\rho}_{\boldsymbol{PNIPAAm}}\boldsymbol{\cdot}{\boldsymbol{wt.}\%}_{\boldsymbol{PNIPAAm}}\boldsymbol{+}\boldsymbol{\rho}_{\boldsymbol{SPIONs}}\boldsymbol{\cdot}{\boldsymbol{wt.}\%}_{\boldsymbol{SPIONs}}$	(5)

Considering that ***ρ_PNIPAAm_*** at 37 °C was estimated to be ≈ 0.90 g·cm^-3^ by Choi *et al.* [12], the density of iron oxide is 5.17 g·cm^-3^ according to [americanelements.com website] and that their weight percentages are 98.78 and 1.22 %, respectively, ***ρ_MINP_*** = 0.95 g·cm^-3^.

The volume of a single MINP was approximated to the volume of a sphere, as given by Equation 6

$\boldsymbol{v}_{\boldsymbol{MINP}}=\frac{\pi}{6}\boldsymbol{\cdot}{\boldsymbol{d}_{\boldsymbol{MINP}}}^{\boldsymbol{3}}$	(6)

with ***d_MINP_*** being the average diameter of MINPs (nm). Since this value was different for each type of MINP, calculations were performed separately for each type.

Therefore, substituting ***ρ_MINP_*** and Equation 6 into Equation 4,

$$m_{MINP}={(\rho}_{PNIPAAm}\cdot{wt.\%}_{PNIPAAm}+\rho_{SPIONs}\cdot{wt.\%}_{SPIONs})\frac{{10}^{3} kg\cdot m^{-3}}{1 g\cdot{cm}^{-3}}\cdot(\frac{\pi}{6}\cdot{d_{MINP}}^{3})\cdot\frac{{10}^{-27} m^{3}}{1 {nm}^{3}}=$$

$m_{MINP}=0.95 \times\frac{\pi}{6}\cdot{d_{MINP}}^{3}\times{10}^{-24} kg$	(7)

Then, substituting Equation 7 into 3, and Equation 3 into 2,

$F_{M}=M\cdot(0.95\times\frac{\pi}{6}\cdot{d_{MINP}}^{3}\times{10}^{-24})\cdot\nabla B$	(8)

In order to estimate the values of *M* according to the |*B*| values provided by FEMM simulations, magnetometry results were fitted to the model in Equation 9,

$$M=\frac{M_{S}\cdot\left| B \right|}{\alpha+\left| B \right|}+\beta\cdot\left| B \right|+\gamma$$

(9)

where *M_S_* (saturation magnetization), *α*, *β*, and *γ* are parameters derived from the fitting. Errors using this model were below 2 % when applied to |*B*| values within the parameters used in FEMM simulations. Moreover, *M_S_* was also close to the experimental value measured by magnetometry for CaPINS (0.7668 emu g^‑1^ vs 0.7391 emu g^-1^, respectively, error of 3.75 %). Finally, substituting Equation 9 with the corresponding parameters into Equation 8, the magnetic force exerted on a single MINP according to its XY position (*F_M_* [N]) can be calculated by Equation 10:

$$F_{M}=\left( \frac{0.7668\times\left| B \right|}{70.93+\left| B \right|}-4.889\times{10}^{-6}\times\left| B \right|-6.676\times{10}^{-4} \right)\cdot\left( 0.95\times\frac{\pi}{6}\cdot{d_{MINP}}^{3}\times{10}^{-24} \right)\cdot\nabla B$$

(10)

|*B*| being the absolute value of the magnetic induction at each XY position as calculated in FEMM simulations (T), *d_MINP_* being the average diameter of a specific MINP type (nm), and ∇*B* being the gradient of magnetic induction (mT·mm^‑1^).

*Estimation of magnetic torque and equivalent force applied to nanoswitches:* Magnetic actuation of receptor-bound nanoparticles was alternatively quantified by estimating the magnetic torque (τ_max_) generated on individual MINPs with incorporated SPIONs, according to Equation 11:

$\tau_{max}=\mu Bsin\theta$	(11)

Assuming that *B* and *μ* are perpendicular (thus, sin*θ* = 1), an average amplitude of $B=467$ mT according to previous simulations or an average of *B* = 371 mT as measured directly with a gaussmeter; and $\mu=3.38\times{10}^{-18}$ A·m^2^ per nanoparticle, as previously calculated, the maximum magnetic torque exerted on a nanoparticle was estimated as:

$\tau_{max}=1.25 \sim1.58\times{10}^{-18}$ N·m.

To express this torque as an equivalent force applied to a receptor, the torque was divided by a molecular lever arm $L$ corresponding to the characteristic length scale of PIEZO1 extracellular domains, as per Equation 12. In the central Cap region, we assumed *L* to be 4–8 nm (roughly the Cap dimensions in the vertical and horizontal axes), while Loop 19-20 is compatible with an *L* of 7-15 nm, as part of the blade domain [13].

$F_{eq}=\frac{\tau_{max}}{L}$	(12)This results in an upper-bound force estimate in the range $F_{eq}\approx1.6\times{10}^{-10}$ to $4.0\times{10}^{-10}$ N (160 – 400 pN) per CaPINS nanoparticle and $F_{eq}\approx0.83\times{10}^{-10}$ to $2.3\times{10}^{-10}$ N (83 – 230 pN) per LooPINS nanoparticle. These values represent the maximum available mechanical load; the effective force transmitted to the channel is expected to be considerably lower due to molecular compliance of the “ligand-receptor” (nanoparticle-channel) complex and local membrane deformation.

To verify that magnetic actuation was not limited by hydrodynamic drag at the stimulation frequency (2 Hz), the viscous torque resisting nanoparticle rotation was estimated by Equation 13:

$\tau_{drag}=8\pi\eta r^{3}\omega$	(13)

where $\eta={10}^{-3}$ Pa·s is the viscosity of the medium, $r=115$ nm was used as the average MINP radius, and $\omega=2\pi f$ with $f=2$Hz. This yielded $\tau_{drag}\approx3\times{10}^{-22}$ N·m, which is four orders of magnitude lower than the magnetic torque. Thus, under the described experimental conditions, nanoparticle rotation should not be limited by viscous drag.

**Supplementary references**

[1]	“ExPASy PeptideCutter,” can be found under https://web.expasy.org/peptide_cutter/.

[2]	E. Gasteiger, C. Hoogland, A. Gattiker, S. Duvaud, M. R. Wilkins, R. D. Appel, A. Bairoch, in The Proteomics Protocols Handbook. Springer Protocols Handbooks. (Ed.: John M. Walker), Humana Press, **2005**, pp. 571–607.

[3]	“Bethesda (MD): National Library of Medicine (US), National Center for Biotechnology Information, BLAST,” can be found under https://blast.ncbi.nlm.nih.gov/Blast.cgi.

[4]	A. Bateman, M.-J. Martin, S. Orchard, M. Magrane, S. Ahmad, E. Alpi, E. H. Bowler-Barnett, R. Britto, H. Bye-A-Jee, A. Cukura, P. Denny, T. Dogan, T. Ebenezer, J. Fan, P. Garmiri, L. J. da Costa Gonzales, E. Hatton-Ellis, A. Hussein, A. Ignatchenko, G. Insana, R. Ishtiaq, V. Joshi, D. Jyothi, S. Kandasaamy, A. Lock, A. Luciani, M. Lugaric, J. Luo, Y. Lussi, A. MacDougall, F. Madeira, M. Mahmoudy, A. Mishra, K. Moulang, A. Nightingale, S. Pundir, G. Qi, S. Raj, P. Raposo, D. L. Rice, R. Saidi, R. Santos, E. Speretta, J. Stephenson, P. Totoo, E. Turner, N. Tyagi, P. Vasudev, K. Warner, X. Watkins, R. Zaru, H. Zellner, A. J. Bridge, L. Aimo, G. Argoud-Puy, A. H. Auchincloss, K. B. Axelsen, P. Bansal, D. Baratin, T. M. Batista Neto, M.-C. Blatter, J. T. Bolleman, E. Boutet, L. Breuza, B. C. Gil, C. Casals-Casas, K. C. Echioukh, E. Coudert, B. Cuche, E. de Castro, A. Estreicher, M. L. Famiglietti, M. Feuermann, E. Gasteiger, P. Gaudet, S. Gehant, V. Gerritsen, A. Gos, N. Gruaz, C. Hulo, N. Hyka-Nouspikel, F. Jungo, A. Kerhornou, P. Le Mercier, D. Lieberherr, P. Masson, A. Morgat, V. Muthukrishnan, S. Paesano, I. Pedruzzi, S. Pilbout, L. Pourcel, S. Poux, M. Pozzato, M. Pruess, N. Redaschi, C. Rivoire, C. J. A. Sigrist, K. Sonesson, S. Sundaram, C. H. Wu, C. N. Arighi, L. Arminski, C. Chen, Y. Chen, H. Huang, K. Laiho, P. McGarvey, D. A. Natale, K. Ross, C. R. Vinayaka, Q. Wang, Y. Wang, J. Zhang, *Nucleic Acids Res* **2023**, *51*, D523.

[5]	J. Jumper, R. Evans, A. Pritzel, T. Green, M. Figurnov, O. Ronneberger, K. Tunyasuvunakool, R. Bates, A. Žídek, A. Potapenko, A. Bridgland, C. Meyer, S. A. A. Kohl, A. J. Ballard, A. Cowie, B. Romera-Paredes, S. Nikolov, R. Jain, J. Adler, T. Back, S. Petersen, D. Reiman, E. Clancy, M. Zielinski, M. Steinegger, M. Pacholska, T. Berghammer, S. Bodenstein, D. Silver, O. Vinyals, A. W. Senior, K. Kavukcuoglu, P. Kohli, D. Hassabis, *Nature* **2021**, *596*, 583.

[6]	M. Varadi, S. Anyango, M. Deshpande, S. Nair, C. Natassia, G. Yordanova, D. Yuan, O. Stroe, G. Wood, A. Laydon, A. Žídek, T. Green, K. Tunyasuvunakool, S. Petersen, J. Jumper, E. Clancy, R. Green, A. Vora, M. Lutfi, M. Figurnov, A. Cowie, N. Hobbs, P. Kohli, G. Kleywegt, E. Birney, D. Hassabis, S. Velankar, *Nucleic Acids Res* **2022**, *50*, D439.

[7]	F. Canfarotta, A. Poma, A. Guerreiro, S. Piletsky, *Nat Protoc* **2016**, *11*, 443.

[8]	S. P. B. Teixeira, A. Pardo, S. M. Bakht, M. Gomez‐Florit, R. L. Reis, M. E. Gomes, R. M. A. Domingues, *Adv Funct Mater* **2024**, *34*, 2312961.

[9]	Y. Hoshino, T. Kodama, Y. Okahata, K. J. Shea, *J Am Chem Soc* **2008**, *130*, 15242.

[10]	K. Ogawa, A. Nakayama, E. Kokufuta, *Langmuir* **2003**, *19*, 3178.

[11]	D. C. Meeker, “Finite Element Method Magnetics (FEMM) 4.2 (28Feb2018 Build)”, can be found under https://www.femm.info, **2018**.

[12]	J. G. Choi, H. Gwac, Y. Jang, C. Richards, H. Warren, G. Spinks, S. J. Kim, *Micromachines (Basel)* **2021**, *12*, 210.

[13]	K. Saotome, S. E. Murthy, J. M. Kefauver, T. Whitwam, A. Patapoutian, A. B. Ward, *Nature* **2018**, *554*, 481.

[14]	P. Blümler, F. Raudzus, F. Schmid, *Sci. Rep.* **2024**, *14*, 7879.
